# Online course on vaccinating people with HIV/AIDS - effectiveness in the knowledge of nursing professionals*

**DOI:** 10.1590/1518-8345.7004.4278

**Published:** 2024-08-12

**Authors:** Larissa Gerin, Elucir Gir, Lis Aparecida de Souza Neves, Luzia Márcia Romanholi Passos, Renato de Ávila Kfouri, Renata Karina Reis

**Affiliations:** 1 Secretaria Municipal da Saúde, Divisão de Vigilância Epidemiológica, Ribeirão Preto, SP, Brazil; 2 Universidade de São Paulo, Escola de Enfermagem de Ribeirão Preto, PAHO/WHO Collaborating Centre for Nursing Research Development, Ribeirão Preto, SP, Brazil; 3 Secretaria Municipal da Saúde, Departamento de Vigilância em Saúde, Ribeirão Preto, SP, Brazil; 4 Sociedade Brasileira de Imunizações, São Paulo, SP, Brazil

**Keywords:** Vaccination Coverage, Vaccination, HIV, Knowledge, Nurse Practitioners, Health Education

## Abstract

**Objective:**

analyzing the effectiveness of an educational intervention on the knowledge of nursing professionals regarding the immunization of people with the human immunodeficiency virus.

**Method:**

a quasi-experimental study evaluated professionals’ knowledge through a knowledge test applied before and after the development of an online training course. The data was analyzed using frequency, median, mean, standard deviation, and association tests.

**Results:**

the sample consisted of 77 nursing professionals whose mean age was 43.2 years (SD+/-8.2). More than half of the individuals worked in basic health units (58.4%), 22.1% worked in specialized services that provide clinical monitoring for people with the human immunodeficiency virus, and 42 (54.5%) were nursing assistants or technicians. The professionals’ performance improved after the intervention, with an increase in the median number of correct answers from 23.0 to 27.0 (p<0.001).

**Conclusion:**

offering an online training course on the immunization of people with the human immunodeficiency virus, as a continuing education activity, proved to be effective in improving nursing professionals’ knowledge on this subject.

## Highlights

**(1)** Services do not evaluate the vaccination status of people living with HIV.

**(2)** The knowledge of health professionals may influence vaccination rates.

**(3)** Health professionals’ knowledge of immunization may be insufficient.

## Introduction

Vaccination is an important public health strategy for preventing infectious diseases in the general population. Over the years, in addition to controlling diseases such as measles and polio, it has been responsible for eradicating smallpox.

For people living with the human immunodeficiency virus (PLHIV), who after the advent of antiretroviral therapy (ART) live in a similar way to uninfected people and are therefore more exposed, it is essential that they have their vaccination schedule updated in accordance with current recommendations, improving quality of life and life expectancy^1^ ^), (^ ^2^ ^), (^ ^3^.

Despite the importance of updating the vaccination schedule for this population, there is a concern on the part of both health professional and the individual himself about the safety and efficacy of the vaccines administered, mainly due to the risk of adverse events based on the degree of immunodepression. In addition, the constant updating of the schedule and the inclusion of new immunizers in the vaccination calendar can generate doubts among health professionals, including nursing professionals who work in vaccination rooms^4^ ^), (^ ^5^.

Delaying the administration of vaccines until the immune system of PLHIV has been completely rebuilt can increase the risk of these individuals, therefore it is recommended that the vaccination schedule be updated early^3^ ^), (^ ^6^ ^), (^ ^7^ ^), (^ ^8^ ^), (^ ^9^ ^), (^ ^10^ ^), (^ ^11^. Although administering some vaccines can generate a transient increase in viral load, this event is not clinically significant and should not prevent vaccination^5^.

PLHIV have an increased risk of acquiring vaccine-preventable diseases and, once they acquire these diseases, they have a greater chance of developing more serious conditions. Because of this scenario, this public has a specific recommendation for vaccination^5^.

In Brazil, the Ministry of Health, through the National Immunization Program (NIP), offers a special vaccination schedule, free of charge, where the individual’s immune status should be evaluated and, if there is no contraindication, the recommendation is that the schedule be updated^1^ ^), (^ ^12^ ^), (^ ^13^.

Although the Ministry of Health does not routinely evaluate vaccination coverage in adults, it is estimated that adherence to the schedule is low in this population. Among PLHIV, this reality is no different, and despite the importance of vaccination in these individuals, studies indicate low vaccination coverage^6^ ^), (^ ^8^ ^), (^ ^10^ ^), (^ ^14^.

A study carried out in the USA showed that less than 50% of PLHIV evaluated were vaccinated appropriately according to the recommendations in force in the country^8^. In another study carried out in Belgium, hepatitis B vaccine coverage in PLHIV was 24.4% and against pneumococcus was 72.6%^14^.

Many factors influence the decision to vaccinate or not, including people, behavior and investment; one of the ways to motivate people to get vaccinated is the health professional’s recommendation^15^ ^), (^ ^16^.

The lack of knowledge among health professionals, especially nursing professionals, regarding the vaccination schedule indicated for PLHIV and all the factors that involve immunizing this public, leads to a lack or insufficiency of recommendations regarding the importance of vaccination, as in addition to collaborating with the individual’s insecurity in getting vaccinated, and the studies that address this issue are scarce^17^ ^), (^ ^18^.

Health professionals, when provided with adequate and quality information on immunization, can provide up-to-date and reliable guidance, which can help to increase confidence in vaccines, in addition, they can monitor the attendance of individuals at the vaccination room and seek out those who are overdue^10^ ^), (^ ^16^.

To this end, continuing health education, through educational actions that take place in everyday work, is an important strategy for increasing the resolution capacity and efficiency of health services with the possibility of causing changes in the social context^19^ ^), (^ ^20^. Nursing professionals need to be constantly trained in content related to immunization.

In view of the above, this study aimed to analyze the effectiveness of an educational intervention on the knowledge of nursing professionals regarding the immunization of people with the human immunodeficiency virus.

## Method

### Study design

This is a quasi-experimental, non-randomized, before-and-after study in which an online educational intervention related to PLHIV immunization was carried out for nursing professionals. The text of this manuscript was developed by the recommendations of SQUIRE 2.0.

### Location of data collection

The study was carried out in the municipality of Ribeirão Preto, São Paulo-SP, Brazil, 312 km from the capital, with an estimated population of 711,825 inhabitants. The municipality has 50 basic health units (BHU) in its public network, 36 of which have a vaccination room, and five specialized services for people living with HIV/AIDS. As for the specialized services, one of them has a vaccination room, three operate in the same physical area as a BHU with vaccination rooms and one of them does not have a vaccination room in the same physical space^21^.

### Population and sample

An e-mail was sent to the supervisors of the 36 health units with vaccination rooms and the 5 specialized services in the municipality’s public network informing them of the start of the intervention phase, which consisted of the online training course. The supervisors were asked to inform the number of professionals interested in taking part in the study by taking the course and filling in the questionnaire applied before and after the course.

Once the number of interested parties had been informed, the units were sent the free and informed consent forms (FICF), and the professionals returned the signed FICF to the researcher, informing her of their contact e-mail address, where they were sent the instructions for registering and accessing the training course.

The sample for this study was therefore intentional, made up of nursing professionals (nursing assistants/technicians and nurses) who agreed to take part in the study and who met one of the following inclusion criteria: working in the vaccination rooms of the municipal public health system, being directly or indirectly involved in municipal public immunization actions at the time the course was offered or working in the specialized services of the Unified Health System (*Sistema* Único *de Saúde*, SUS) in the municipality of the study where PLHIV are monitored.

### Educational intervention

An online training course was developed through the Virtual Learning Environment (VLE) Moodle, in the form of an extension course, provided by the University of São Paulo (USP), entitled “Training on immunization of people living with acquired immunodeficiency virus infection - HIV/AIDS”.

The course consisted of four modules and was designed by the NIP guidelines^12^ and the scientific literature on the subject. The educational program was built on the principles of andragogy, taking into account the learning needs reported by those invited to take part in the course, based on the researcher’s experience as coordinator of the immunization program and epidemiological surveillance nurse, and the difficulties perceived over time. By the methodological proposal of andragogy, the learning process was self-directed, in an informal and collaborative atmosphere between students and educator^22^.

The course was offered asynchronously between August and November 2021, lasted a total of three hours, and was available for access according to the participant’s availability. The content was divided into four modules: 1 - Importance of vaccination as a public health practice/significance of health promotion, 2 - Basic concepts in immunization, 3 - Attenuated and inactivated vaccines - indications for PLHIV and 4 - Vaccination schedule for HIV-infected adults.

The course consisted of a recorded lesson, a discussion forum for questions and support material (bibliographical reference and slides used in the lesson) to facilitate understanding.

In accordance with the proposed timetable, from December/2021 it was no longer possible to enroll in the course. Since classes and content must be updated in line with current recommendations, the course was no longer made available.

### Instrument used to collect information

The data used to evaluate knowledge was collected on the Moodle platform where the course was offered, using a questionnaire called knowledge test, drawn up based on NIP guidelines^12^ by a professional with extensive experience in the field of immunization. The data collection instrument was evaluated by six specialists with professional experience in the areas of immunization and HIV, all of whom were nurses, masters, and doctors.

The experts evaluated the questionnaire in terms of content, objective, verbal language and relevance. The Content Validity Index (CVI) was calculated for each item using a five-point scale, based on the set of evaluation characteristics: content (9 items; CVI=1.00), objective (5 items; CVI=0.97), verbal language (7 items; CVI: 0.95), relevance (3 items; CVI=1.00) and the total instrument (24 items; CVI=0.97). All the judges’ suggestions were accepted.

The knowledge test had two parts, one with 10 questions about the participants’ identification data and the other with 35 questions about the professionals’ technical knowledge about immunizing PLHIV. The questions that evaluated the professionals’ technical knowledge were divided into four categories according to the topics covered in the training course modules: I. Importance of vaccination as a public health practice/significance of health promotion (11 questions); II. Basic concepts in immunization (6 questions); III. Attenuated and inactivated vaccines - indications for PLHIV (8 questions); IV. Vaccination schedule for HIV-infected adults (10 questions). Objective questions were used and for each of them one of the following options had to be chosen: “I agree”, “I disagree” or “I don’t know”, with only one being the correct answer.

### Study variables

The study variables were those related to the participants’ identification - sex, age, place of work, length of time working, and data regarding education; and those related to technical knowledge on immunization of PLHIV addressed in each category of knowledge evaluated.

### Data collection

The training course that made up the educational intervention in this study was held from August to November 2021.

Data was collected by filling in the knowledge test on the platform where the course was held, before and after the training course was developed.

### Data processing and analysis

After completing the online training course, the data was extracted from the course platform in a Microsoft Office Excel^®^ spreadsheet and transferred to the IBM^®^ Statistical Package for the Social Sciences (SPSS^®^) Statistics version 25 program, where the data was analyzed. To calculate Cohen’s “d”, the Cohen.d function from the “effsize” package of the R statistic software.

Identification data was described using frequency distribution (absolute and relative), median, mean, and standard deviation. For the knowledge test, the answers were categorized as “correct” and “incorrect”, then a count was made of how many questions each participant answered correctly before (Q1_correct answer variable) and after the intervention (Q2_correct answer variable). The normality of the two variables was tested and only the Q1_correct answer variable showed normality (Shapiro-Wilk test p=0.194).

The course participants’ knowledge was evaluated according to the following concepts determined by a 20% correct answer rate - insufficient (0┤20), regular (20┤40), good (40┤60), optimal (60┤80), excellent (80┤100), as proposed by other authors^23^.

The data was separated into the categories that made up the knowledge test. As they did not pass the normality test, they were compared using the non-parametric Wilcoxon paired test to compare the median number of correct answers before and after the training course for each question, the total number of questions, and the knowledge category.

Spearman’s correlation coefficient (age, length of time working, length of education), Kruskal-Wallis test for independent samples (unit and district in which they work and highest level of education), and Mann-Whitney U-test for independent samples (position held in the Municipal Health Department/MHD and whether they had received training in immunization) and p-values<0.05 were considered as statistical evidence.

### Ethical aspects

The research project was submitted to the Research Project Evaluation Committee (*Comissão de Avaliação de Projetos de Pesquisa*, CAPP) of the MHD of the municipality of the study and then to the Research Ethics Committee (REC) of the Ribeirão Preto Nursing School (*Escola de Enfermagem de Ribeirão Preto*, EERP) of USP, obtaining a favorable opinion (CEP Consubstantiated Opinion No. 4.782.341).

## Results

After the study was publicized in the health units, 143 professionals signed the FICF and received instructions on how to enroll in the course by email. Of these, 130 enrolled on the platform and 90 accessed the content. However, 77 (100%) completed all the stages of the course, making up the sample for this study.

The mean age of the participants was 43.2 years (min-max: 24-69; SD+/-8.2), 75 (97.4%) were female and the most frequent age group was 40 to 49 years (36; 46.7%) (Table 1).

**Table 1:** Distribution of participants according to sex, age group, education and professional activity (n=77). Ribeirão Preto, SP, Brazil, 2021

Characteristics	Participants	
	f*	%
**Sex**		
Male	2	2.6
Female	75	97.4
**Age group (years)**		
20 to 29	2	2.6
30 to 39	23	29.9
40 to 49	36	46.7
50 to 59	14	18.2
60 or more	2	2.6
**Health unit where they work**		
Specialized service 1	5	6.5
Specialized service 2	6	7.8
Specialized service 3	2	2.6
Specialized service 4	1	1.3
Specialized service 5	3	3.9
BHU^†^/Family Health Unit	45	58.4
Emergency Service	6	7.8
Epidemiological Surveillance	3	3.9
Others	6	7.8
**District where they work**		
East	32	41.6
Central	15	19.5
North	10	13.0
West	11	14.3
South	3	3.9
No district	6	7.8
**Length of time working in vaccination room/specialized service (years)**		
0 to 9	46	59.7
10 to 19	23	29.9
20 to 29	7	9.1
30 to 39	1	1.3
**Length of education (years)**		
0 to 9	8	10.4
10 to 19	37	48.0
20 to 29	22	28.6
30 to 39	10	13.0
**Position held in the MHD** ^‡^		
Nursing Assistant/Technician	42	54.5
Nurse	34	44.2
Not an MHD^‡^ employee^‡^	1	1.3
**Immunization training**		
Yes	52	67.5
No	25	32.5

*f=Frequency; ^†^BHU=Basic Health Unit; ^‡^MHD=Municipal Health Department

Among the participants, 32 (41.6%) worked in the eastern district. Regarding their place of work, 17 (22.1%) worked in specialized services that care for PLHIV and 45 (58.4%) worked in BHUs. Overall, 46 (59.7%) professionals had been working in their units for less than 10 years, with a mean time in service of 7.9 years (SD+/-7.7) (Table 1).

The mean length of education was 18.4 years (SD+/-7.4), with a minimum of 2 and a maximum of 35 years of education. Almost half of the participants (37; 48.0%) had graduated between 10 and 19 years ago and 42 (54.5%) were nursing assistants or technicians at the MHD. Among the participants, 52 (67.5%) had previously undergone immunization training (Table 1).

In terms of knowledge, the mean number of correct answers went from 22.6 (SD+/-4.3) in the pre-test to 26.6 (SD+/-4.3) after the training course and performance improved with an increase in the median number of correct answers from 23.0 to 27.0 (p<0.001).

In the pre-test, 38 participants (49.4%) had a number of correct answers below the median (23.0), while in the post-test, 67 participants (87.0%) had a number of correct answers equal to or above this index.

The value obtained for Cohen’s d was - 0.94 (CI=[-1.277; -0.606]). Since the value 0 is not included, there is evidence, at the 95% confidence level, that there is a significant difference between the means, and as presented by Cohen^24^, the value found provides evidence that the magnitude of the effect of this difference is high.

In category 1 - Importance of vaccination as a public health practice/meaning of health promotion, five questions (45.4%) had a correct answer rate of over 80% in the pre-intervention phase. In the post-intervention phase, the “excellent” concept was achieved in eight questions (72.7%).

The mean number of correct answers in this category was 8.3 (SD+/-1.4) in the pre-test, an increase was observed in the post-test with a mean of 9.2 correct answers (SD+/-1.5) and the median number of correct answers went from 8.0 to 9.0 (p<0.001).

An increase in the number of correct answers occurred in eight questions (72.7%). The questions on which professionals can recommend vaccination for PLHIV (Q1) and on the need for a doctor’s prescription to administer vaccines to this public (Q3) showed p<0.001. The question about vaccination coverage in adults (Q6) had p=0.007 and the question about vaccine hesitancy (Q8) had p=0.002. Questions 1, 3, and 8 went from an optimal level of knowledge in the pre-test to an excellent level in the post-test; question 6 went from a regular level to a good level.

The questions that showed that health professionals are aware of the fact that the anti-vaccination movement has been gaining strength in Brazil in recent years, with an increase in the number of correct answers from 88.3% to 94.8% (Q7) and from 71.4% to 77.9% (Q10), showed p=0.132 and p=0.225, respectively (Table 2).

**Table 2:** Comparison between the percentage of correct answers to the knowledge test questions in category 1 (Importance of vaccination as a public health practice) before and after the training course (n=11, 100%). Ribeirão Preto, SP, Brazil, 2021

Nº	Category 1 questions	Before	After	p-value*
		% of correct answers	% of correct answers	
Q1	The only health professional responsible for recommending vaccines to PLHIV^†^ is the infectologist. (D^‡^)	64.9	93.5	**<0.001**
Q2	PLHIV^†^ have specific vaccination recommendations from the NIP^§^. (A^||^)	96.1	97.4	0.655
Q3	The Vaccination Room team can only administer vaccines to PLHIV^†^ that have been prescribed by the infectologist who is monitoring the patient. (D^‡^)	66.2	94.8	**<0.001**
Q4	The active search for individuals with an overdue vaccination schedule by the team from the Vaccination Rooms and Specialized Services is an important action to guarantee the completeness of the vaccination schedule. (A^||^)	**100.0**	**96.1**	0.083
Q5	Some diseases have already been controlled, such as measles, so in the risk-benefit evaluation, there is no need to administer the measles vaccine to PLHIV^†^. (D^‡^)	85.7	89.6	0.366
Q6	The NIP^§^ makes various immunizers available through the SUS^¶^ and the country maintains good vaccination coverage in adults. (D^‡^)	24.7	44.2	**0.007**
Q7	Many countries face problems with vaccine refusal, a complex phenomenon that involves several factors, and this phenomenon has been established in Brazil in recent years. (A^||^)	88.3	94.8	0.132
Q8	Individuals who do not agree to complete the vaccination schedule, but do agree to receive some vaccines, can be considered vaccine hesitant. (A^||^)	76.6	93.5	**0.002**
Q9	As the number of vaccines offered and their use through vaccination programs increases, people’s concern about the safety of immunizers and distrust of the need for their use decreases. (D^‡^)	**57.1**	**37.7**	**0.014**
Q10	Anti-vaccination movements began in the 19^th^ century with the use of smallpox vaccine, the first vaccine developed, and have been gaining strength in recent years due to their spread on social media. (A^||^)	71.4	77.9	0.225
Q11	The drop in vaccination coverage increases the incidence of preventable diseases, and consequently increases the number of preventable deaths, which is a public health risk. (A^||^)	**100.0**	**98.7**	0.317

*The significance level is 0.05; ^†^PLHIV=People living with the human immunodeficiency virus; ^‡^D=Disagree; ^§^NIP=National Immunization Program; ^||^A=Agree; ^¶^SUS=Unified Health System

Regarding the basic immunization concepts evaluated in category 2, two questions (33.3%) obtained a correct answer rate higher than 80% in the pre-intervention phase. In the post-intervention phase, this increased to three questions (50.0%) with the concept “excellent”. The mean of 3.6 correct answers (SD+/-1.3) in the pre-test increased to 4.4 correct answers (SD+/-1.3) in the post-test and the median number of correct answers remained at 4.0 (p=<0.001).

After the educational intervention, four questions (66.7%) showed an increase in the number of correct answers. The question evaluating the composition of vaccines (Q13) had p<0.001 and went from insufficient in the pre-test to a good level of correct answers in the post-test; and the question addressing the responsiveness of the unconjugated polysaccharide vaccine (Q14) had p<0.001 and went from a regular level of knowledge in the pre-test to excellent in the post-test (Table 3).

**Table 3:** Comparison between the percentage of correct answers to the knowledge test questions in category 2 (Basic concepts in immunization) before and after the training course (n=6, 100%). Ribeirão Preto, SP, Brazil, 2021

Nº	Category 2 questions	Before	After	p-value*
		% of correct answers	% of correct answers	
Q12	Combination vaccines are those in which an immunologically less potent product is added to another immunologically more potent product, thus enabling the first product to acquire characteristics of immunological potency that it did not previously possess. (D^†^)	42.9	53.2	0.131
Q13	A conjugate vaccine is one made up of two or more antigens from different infectious agents in a single preparation. (D^†^)	18.2	44.2	**<0.001**
Q14	In inactivated polysaccharide non-conjugated vaccines, immunity is short-lived (three to five years, in general), as the immune response does not involve stimulation of lymphocytes related to cellular immunity. (A^‡^)	39.0	85.7	**<0.001**
Q15	During pre-vaccination screening, possible contraindications should be investigated, for example, the use of antibiotics, which contraindicates most vaccines. (D^†^)	89.6	94.8	0.248
Q16	It is recommended to postpone vaccination in case of moderate or severe acute febrile illness until the condition improves. (A^‡^)	**98.7**	**97.4**	0.564
Q17	The occurrence of adverse events must be reported, any adverse event constitutes a contraindication for future doses. (D^†^)	**74.0**	**68.8**	0.317

*The significance level is 0.05; ^†^D=Disagree; ^‡^A=Agree

For the questions that presented the definition of combined vaccines (Q12) and the situation of contraindication for administering vaccines (Q15), the increase in the number of correct answers was from 42.9% to 53.2% and 89.6% to 94.8%, with p=0.131 and p=0.248, respectively (Table 3).

Category 3 evaluated attenuated and inactivated vaccines - indications for PLHIV. It was found that four questions (50.0%) obtained a correct answer rate above 80% in the pre-test, and five (62.5%) in the post-test.

In the pre-test the mean was 5.5 correct answers (SD+/-1.3) in this category and increased in the post-test with a mean of 6.3 correct answers (SD+/-1.4), the median went from 6.0 to 7.0 (p<0.001).

The increase in the number of correct answers occurred in five questions (62.5%). The question on the composition of the hepatitis A vaccine (Q18) had p=0.035, the question on the administration of live attenuated vaccines among PLHIV (Q19) with p<0.001, and the questions on the interval between inactivated vaccines (Q21and Q23) with p=0.050 and p=0.001, respectively (Table 4).

**Table 4:** Comparison between the percentage of correct answers to the knowledge test questions in category 3 (Attenuated and inactivated vaccines - indications for PLHIV*) before and after the training course (n=8, 100%). Ribeirão Preto, SP, Brazil, 2021

Nº	Category 3 questions	Before	After	p-value^†^
		% of correct answers	% of correct answers	
Q18	The hepatitis A vaccine is made up of inactivated virus. (A^‡^)	85.7	94.8	**0.035**
Q19	Live attenuated viral vaccines prepared with live antigens cannot be administered to PLHIV*. (D)	42.9	77.9	**<0.001**
Q20	Inactivated vaccines are produced from inactivated whole microorganisms or particles of microorganisms and are not capable of producing disease. (A^‡^)	**94.8**	**93.5**	0.317
Q21	A health service user attends to receive the Double Adult and Triple Viral vaccines with a referral from the Specialized Service professional. The vaccination room professional notes that the user received a dose of ACWY meningococcal vaccine in the private network 10 days ago and advises the patient to return in 20 days, as it is contraindicated to do other vaccinations with an interval of less than 30 days. (D^§^)	67.5	79.2	**0.050**
Q22	Inactivated vaccines are generally not contraindicated for PLHIV*. (A^‡^)	**83.1**	**81.8**	0.819
Q23	A 30-day interval must be observed between inactivated vaccines when not administered on the same day. (D^§^)	61.0	84.4	**0.001**
Q24	Attenuated vaccines can only be administered to asymptomatic PLHIV* with a CD4 T lymphocyte count>350 cells/mm^3^. (D^§^)	22.1	23.4	0.847
Q25	COVID-19 vaccines that behave like inactivated vaccines can be administered to PLHIV*. (A^‡^)	**96.1**	**94.8**	0.655

*PLHIV=People living with the human immunodeficiency virus; ^†^Significance level is 0.05; ^‡^A=Agree; ^§^D=Disagree

Question 18 remained at the excellent knowledge level, question 19 went from the good level in the pre-test to the optimal level in the post-test, question 21 remained at the optimal level and question 23 went from the optimal level to the excellent level (Table 4).

Question 24, which evaluated knowledge about administering attenuated vaccines to asymptomatic PLHIV with CD4 T-lymphocyte (TL) counts >350 cells/mm^3^, had a regular level of knowledge before and after the intervention (Table 4).

Concerning the vaccination schedule for adult PLHIV, evaluated in category 4, of the ten questions prepared, only one (10.0%) obtained a correct answer rate of more than 80% in the test applied before the training course; in the post-test, three questions (30.0%) obtained the concept “excellent”.

The mean number of correct answers in the pre-test was 5.2 (SD+/-1.9) and 6.7 (SD+/-1.6) in the post-test, with the median number of correct answers rising from 5.0 to 7.0 (p<0.001).

There was an increase in the number of correct answers in nine questions (90.0%). Only the question that dealt with the meningococcal C vaccine schedule (Q31) had the lowest number of correct answers both before and after the training course, and there was also a decrease in the number of correct answers, from a regular level of knowledge in the pre-test to an insufficient level in the post-test, with p=0.317 (Table 5).

**Table 5:** Comparison between the percentage of correct answers to the knowledge test questions in category 4 (Vaccination schedule for adults infected with the human immunodeficiency virus) before and after the training course (n=10, 100%). Ribeirão Preto, SP, Brazil, 2021

Nº	Category 4 questions	Before	After	p-value*
		% of correct answers	% of correct answers	
Q26	Influenza vaccine is contraindicated for people with CD4 T lymphocytes<200 cells/mm^3^. (D^†^)	57.7	74.0	**0.009**
Q27	PLHIV^‡^ are recommended to receive pneumococcal vaccine 23 in a two-dose schedule with a 5-year interval between doses. (A^§^)	72.7	93.5	**0.000**
Q28	It is contraindicated to administer the yellow fever vaccine to PLHIV^‡^. (D^†^)	54.5	81.8	**0.000**
Q29	Pneumococcal vaccine 23 should be administered to PLHIV^‡^ 8 weeks after receiving pneumococcal vaccine 13. (A^§^)	51.9	63.6	**0.050**
Q30	The monovalent varicella vaccine is indicated for all PLHIV^‡^. (D^†^)	44.2	62.3	**0.011**
Q31	The vaccination schedule with the meningococcal C vaccine for PLHIV^‡^ is two doses with a 60-day interval. (D^†^)	**23.4**	**18.2**	0.317
Q32	After completing the hepatitis B vaccination regimen for PLHIV^‡^, serology is recommended to evaluate seroconversion. If the serology is negative, it is recommended to repeat the vaccination schedule. (A^§^)	55.8	72.7	**0.024**
Q33	Vaccinating health professionals and family members of PLHIV^‡^ is a way of increasing protection for this public. (A^§^)	93.5	100.0	**0.025**
Q34	The ideal time to start any vaccine for PLHIV^‡^ is 6 months after starting ART^||^. (D^†^)	40.3	58.4	**0.013**
Q35	The HPV^¶^ vaccination schedule for PLHIV^‡^ is three doses (0, 2 and 6 months) in the 9-26 age group. (D^†^)	16.9	48.1	**0.000**

*The significance level is 0.05; ^†^D=Disagree; ^‡^PLHIV=People living with the human immunodeficiency virus; ^§^A=Agree; ^||^ART=Antiretroviral Therapy; ^¶^HPV=Human Papillomavirus

On the other hand, it should be noted that six (60.0%) of the questions in this category obtained a good level of knowledge in the pre-test, and four of them (66.7%) achieved an excellent level in the post-test. This was the category with the greatest increase in the level of knowledge among the participants after the training course (Table 5).

The median number of correct answers improved in all the categories evaluated, all with p<0.001, and was higher in categories 1 (Importance of vaccination as a public practice/significance of health promotion), 3 (Attenuated and inactivated vaccines - indications for PLHIV) and 4 (Vaccination schedule for HIV-infected adults). In category 2 (Basic concepts in immunization), the median number of correct answers remained the same, but the mean number of correct answers was higher in the post-test.

In the comparison with work and education characteristics, the highest number of correct answers was associated with the unit in which they work in the pre-test (p=0.038), with a higher mean number of correct answers among Epidemiological Surveillance professionals (27.0) and Specialized Service professionals 1 (26,0).

## Discussion

The educational intervention developed proved to be effective in improving the knowledge of nursing professionals about the immunization of PLHIV. This result is relevant and validates the use, by managers, of a similar intervention as part of continuing education to improve and develop the broad knowledge base of nursing professionals on this subject.

In the literature, another study also showed that short, targeted online training courses were effective in improving knowledge of specific health-related content and could be an option in clinical practice, collaborating in continuing education^25^.

Continuing health education through an educational intervention directed at nursing professionals has proven to be an effective strategy for improving their knowledge and confidence about immunization of PLHIV, which can have a positive impact on vaccination coverage among this public.

It is worth noting that no study in the literature was found on the specific context of health professionals’ knowledge related to immunization of PLHIV^17^. A study carried out with nurses in Qatar identified an improvement in the knowledge of vaccination in Primary Health Care nursing after a training course and significant gaps were found in different aspects^26^.

The nursing professionals had good knowledge of most of the aspects evaluated, even before the training course, but several gaps were identified, which could be overcome after the course with an improvement in the median number of correct answers.

In the knowledge test, there was an increase in the number of correct answers to the question that stated that only an infectologist could recommend vaccines for PLHIV. This question is important, since any trained health professional can evaluate vaccination status and recommend vaccines, which helps to achieve the coverage needed to control infectious diseases, especially in this population at high risk of complications from these diseases^12^ ^), (^ ^13^ ^), (^ ^14^ ^), (^ ^27^ ^), (^ ^28^ ^), (^ ^29^.

There was also an increase in knowledge about the need for a doctor’s prescription for vaccinating PLHIV. According to the protocols, only attenuated vaccines need a doctor’s prescription for this group, as they are contraindicated in the presence of severe immunodepression^12^ ^), (^ ^13^ ^), (^ ^27^ ^), (^ ^30^.

Most of the professionals taking part in this study agreed that vaccine hesitancy has been gaining ground in Brazil in recent years. More than 70%, both before and after the training course, agreed that the anti-vaccination movement has been gaining strength in the country. In the pre-test, all participants agreed that the drop in vaccination coverage is a risk to public health as it increases the occurrence of preventable diseases and, consequently, the number of deaths.

Since 2016, vaccination coverage has been falling in Brazil for children, and the country has seen an increase in vaccine hesitancy. Despite not evaluating coverage in adults, it is estimated that this scenario is also reflected in this population. Concerning PLHIV, there is no effective evaluation of their vaccination status in the services where they are monitored^5^.

With the growth of the anti-vaccination movement, especially after the vaccination campaign against COVID-19, with the spread of fake news and conspiracy theories, the population’s distrust of immunizers has worried health authorities, since the drop in vaccination coverage increases the risk of occurrence and reintroduction of controlled or already eradicated diseases^10^ ^), (^ ^16^ ^), (^ ^31^ ^), (^ ^32^ ^), (^ ^33^ ^), (^ ^34^ ^), (^ ^35^.

More than 80% of the participants in this study disagreed with the statement that it is not necessary to administer vaccines for diseases that are already under control, but less than 50% disagreed that concern about the safety of vaccines and distrust of them has decreased with the increase in the number of immunizers on offer. In reality, as the number of vaccines on offer increases and the diseases are controlled, the fear of adverse events from the vaccine becomes greater than the fear of the disease itself^34^ ^), (^ ^36^.

Less than half of the participants in this study in the pre-test disagreed with the statement that the best time to start vaccinating PLHIV is six months after starting ART, and in the post-test those who disagreed with this statement did not reach 60%, which shows the insecurity of professionals in vaccinating this public.

Even with the possibility of a reduced vaccine response in the presence of uncontrolled viral replication or lower CD4 TL values, it is recommended that vaccines be administered according to the current schedule, as soon as HIV infection is diagnosed, and as soon as possible contraindications for attenuated vaccines have been evaluated according to immune status^2^ ^), (^ ^3^ ^), (^ ^12^ ^), (^ ^13^.

The Brazilian Ministry of Health does not recommend intervals between test collection and vaccine administration, although some studies recommend not measuring viral load in the weeks following vaccination^12^ ^), (^ ^13^ ^), (^ ^37^.

More than 90% of the professionals in this study agreed, both pre- and post-test, that inactivated vaccines are not capable of causing the diseases they immunize against. More than 80% agreed that these vaccines are generally not contraindicated for PLHIV. Although inactivated vaccines are not contraindicated for PLHIV, professionals may still feel insecure about the safety of these vaccines in this population^12^ ^), (^ ^13^ ^), (^ ^28^ ^), (^ ^38^.

Almost all the participants in this study agreed that PLHIV have specific vaccination recommendations, and that the complexity of the schedule for this public requires constant updating, training and qualification of the teams involved.

Topics such as the composition of vaccines generate doubts among health professionals. Despite an increase in the number of correct answers in the post-test, category 2 - Basic concepts in immunization contained the question with one of the worst performances in the knowledge test applied before the training course, which dealt with the definition of conjugate vaccine. Not understanding the composition of vaccines means that professionals also don’t understand the recommendation for each schedule for different immunobiologicals and the need or not for boosters.

Another question with poor performance was related to the meningococcal C vaccine schedule for PLHIV, which in the post-test showed a further deterioration in the number of correct answers, demonstrating that even after the training course the professionals had doubts. Perhaps during the training course, the vaccination schedule was not clearly presented, just as it was not clear in the technical document made available by the NIP at the time^12^. This document was updated in 2023, the meningococcal C vaccine was replaced by the meningococcal ACWY vaccine and the vaccination schedule for this public is defined as two doses with an interval of 8 weeks and boosters every 5 years^13^.

Another factor that makes it difficult for health professionals to assimilate is the different schedules for each age group, which can also be demonstrated by the performance of professionals in the pre-test in relation to the human papillomavirus (HPV) vaccine schedule, which showed the worst result in this phase of the study.

The category of the knowledge test that addressed questions about the importance of vaccination in public health was the best performing, reinforcing that professionals from vaccination rooms and services that monitor PLHIV are aware of the role of immunization in preventing diseases and improving quality of life.

The category with the worst performance was the one addressing the vaccination schedule for PLHIV, showing that even after taking part in the training course they still had doubts about it. This reinforces the importance of continuing health education to reduce the knowledge deficit concerning vaccination schedules for adults, especially those belonging to specific population groups. Greater familiarity with vaccination schedules and the application of immunobiologicals at the place where the individuals are monitored can help to increase vaccination rates^8^ ^), (^ ^9^ ^), (^ ^10^ ^), (^ ^16^ ^), (^ ^19^ ^), (^ ^28^ ^), (^ ^29^ ^), (^ ^31^ ^), (^ ^32^ ^), (^ ^33^ ^), (^ ^39^ ^), (^ ^40^ ^), (^ ^41^.

Although the drop in vaccination coverage is affected by multiple factors, the knowledge of health professionals about the vaccines indicated for PLHIV, their schedules, and their contraindications can have a significant impact on this indicator, as it helps the individual to make a decision about vaccination^34^ ^), (^ ^41^. In addition, this is a subject that is little covered in the literature, which results in insecurity^17^.

Before the training course, around 22% of the professionals disagreed that attenuated vaccines could only be given to asymptomatic PLHIV with a CD4 TL value above 350 cells/mm^3^. The number of correct answers to this question increased little in the post-test, and this was the question with the second worst performance in this phase of the study. Even after the development of the course, around 20% of professionals believed that PLHIV could not receive the yellow fever vaccine.

A study carried out in the USA showed that the CD4 TL count is one of the factors associated with non-adherence to vaccination in PLHIV^8^, although it is known that administering vaccines to PLHIV is a safe, effective, and important strategy in this group, especially inactivated vaccines^28^. In conclusion, it is not clear to health professionals which condition contraindicates immunization of PLHIV.

Regarding the inactivated influenza vaccine, which is recommended for all PLHIV regardless of immune status, especially for those who are immunosuppressed, in the knowledge test there was an increase in the number of correct answers regarding the indication of the immunizer for PLHIV, but in the post-test, around 25% still believed that the vaccine would be contraindicated in the presence of severe immunosuppression.

Health professionals need to know the real contraindications and the situations in which postponement of vaccination is recommended so that they don’t collaborate in maintaining low vaccination coverage. Doctors also need to be careful when prescribing vaccines that can only be administered on presentation of this document^8^ ^), (^ ^13^ ^), (^ ^27^.

To guarantee the protection of PLHIV, especially among those with temporary contraindications to receiving any vaccine, the NIP recommends the administration of certain vaccines to health professionals and to household contacts of this public^13^. This recommendation was already known by 93% of the professionals who took part in this study in the pre-test. After taking part in the training course, all the professionals agreed that this is an important strategy.

The gaps related to immunization must be tackled from the initial education of health professionals, and it is necessary to increase the workload related to immunization, as individuals tend to trust the guidance of health professionals and teams^14^.

Evaluating vaccination status at every visit to the health service, regardless of medical care, can be an effective measure to improve vaccination coverage in this population. To do so, these professionals need to be up-to-date on the subject and confident in their guidelines. In addition, it is necessary to monitor the attendance of individuals at the vaccination service and search for those who are behind in their vaccination schedule^8^ ^), (^ ^42^.

The participants in this study agreed that active search is an important strategy, even before the training course. The reasons for non-vaccination, which leads to low vaccination coverage, need to be known by managers and health professionals so that measures can be put in place to face this growing problem around the world^38^ ^), (^ ^39^.

Despite the importance of this study in reinforcing that health professionals have doubts about the vaccination schedule for PLHIV, which can have a direct impact on vaccination coverage, it is necessary to point out some limitations.

The training course was offered in the middle of the national vaccination campaign against COVID-19, a time of work overload for the teams, which had a direct impact on professional adherence. In addition, the online-only course made it difficult for health professionals who were not trained in the use of this technology to access it. On the other hand, it is believed that taking the course asynchronously online facilitated the participation of professionals who would not have been able to take the course in person at the time. Another limiting factor is that the sample was selected by convenience, which may have generated some bias, since those who agreed to take the training course may have felt more comfortable with the topic.

Despite these limitations, this study presents robust results that reinforce the importance of continuing education in ensuring better-qualified professionals to evaluate the vaccination status of individuals, recommend and administer the vaccines indicated in the NIP protocols, monitor the completeness of the vaccination schedule and search for individuals who are behind in their vaccination schedule.

## Conclusion

The data from this study showed that the knowledge of nursing professionals in the services where PLHIV are monitored and in the vaccination rooms may be insufficient in various aspects, which may contribute to vaccination not being indicated or vaccination opportunities being missed.

The knowledge of health professionals, especially nurses, about the vaccines offered, the recommended vaccination schedules and the contraindications when administering immunobiologicals is one of the factors that can directly influence vaccination coverage.

The continuing education activity carried out by offering an online training course on the immunization of PLHIV proved to be effective since there was an improvement in the knowledge of nursing professionals when comparing the tests applied before and after the development of the course, in addition, the effect size of the intervention was considered high.
