# Supplementary material for: Online course on vaccinating people with HIV/AIDS - effectiveness in the knowledge of nursing professionals
Source: Rev Lat Am Enfermagem. 2024 Aug 12;32:e4278. doi: 10.1590/1518-8345.7004.4278 (PMC11321195; doi:10.1590/1518-8345.7004.4278)
Supplement: Translation

# Translation: Curso *online* sobre vacinação de pessoas com HIV/aids - efetividade no conhecimento de profissionais de enfermagem*

**DOI:** 10.1590/1518-8345.7004.4279

**Authors:** Larissa Gerin, Elucir Gir, Lis Aparecida de Souza Neves, Luzia Márcia Romanholi Passos, Renato de Ávila Kfouri, Renata Karina Reis

**Affiliations:** 1 Secretaria Municipal da Saúde, Divisão de Vigilância Epidemiológica, Ribeirão Preto, SP, Brasil; 2 Universidade de São Paulo, Escola de Enfermagem de Ribeirão Preto, Centro Colaborador da OPAS/OMS para o Desenvolvimento da Pesquisa em Enfermagem, Ribeirão Preto, SP, Brasil; 3 Secretaria Municipal da Saúde, Departamento de Vigilância em Saúde, Ribeirão Preto, SP, Brasil; 4 Sociedade Brasileira de Imunizações, São Paulo, SP, Brasil

**Keywords:** Cobertura Vacinal, Vacinação, HIV, Conhecimento, Profissionais de Enfermagem, Educação em Saúde

## Abstract

**Objetivo:**

analisar a efetividade de uma intervenção educativa no conhecimento de profissionais de enfermagem em relação à imunização de pessoas com o vírus da imunodeficiência humana.

**Método:**

estudo quase-experimental que avaliou o conhecimento dos profissionais por meio de um teste de conhecimento aplicado antes e depois do desenvolvimento de um curso de capacitação online. Os dados foram analisados mediante frequência, mediana, média, desvio-padrão e testes de associação.

**Resultados:**

a amostra foi constituída por 77 profissionais de enfermagem, cuja média de idade foi de 43,2 anos (DP+/-8,2). Mais da metade dos indivíduos trabalhava em unidades básicas de saúde (58,4%), 22,1% trabalhavam nos serviços especializados que realizam o acompanhamento clínico de pessoas com o vírus da imunodeficiência humana, 42 (54,5%) exerciam a função de auxiliar ou técnico de enfermagem. O desempenho dos profissionais melhorou após a intervenção, com aumento na mediana de acertos de 23,0 para 27,0 (p<0,001).

**Conclusão:**

o oferecimento de um curso de capacitação *online* acerca da imunização de pessoas com o vírus da imunodeficiência humana, como uma atividade de educação permanente, se mostrou efetivo para melhorar o conhecimento dos profissionais de enfermagem nesta temática.

## Destaques

**(1)** Os serviços não avaliam a situação vacinal de pessoas com HIV.

**(2)** O conhecimento dos profissionais de saúde pode influenciar as taxas de vacinação.

**(3)** O conhecimento dos profissionais de saúde em imunização pode estar sendo insuficiente.

## Introdução

A vacinação é uma importante estratégia em saúde pública na prevenção de doenças infecciosas na população geral. Ao longo dos anos, além do controle de doenças como o sarampo e a poliomielite, foi responsável pela erradicação da varíola.

As pessoas que vivem com o vírus da imunodeficiência humana (PVHIV), após o advento da terapia antirretroviral (TARV), vivem semelhantemente às pessoas não infectadas, e por isso se expõem mais. Dessa forma, é primordial estar com o seu calendário de vacinação atualizado conforme as recomendações vigentes, melhorando a qualidade e a expectativa de vida^1^ ^), (^ ^2^ ^), (^ ^3^.

Apesar da importância da atualização do esquema vacinal deste público, existe um receio, tanto do profissional de saúde quanto do próprio indivíduo, em relação à segurança e à eficácia das vacinas administradas, em função, principalmente, dos riscos de eventos adversos segundo o grau de imunodepressão. Ademais, as constantes atualizações do esquema e a inclusão de novos imunizantes no calendário vacinal podem gerar dúvidas entre os profissionais de saúde, incluindo os profissionais de enfermagem que atuam em salas de vacinas^4^ ^), (^ ^5^.

Retardar a administração de vacinas até a reconstrução completa do sistema imune das PVHIV pode aumentar o risco destes indivíduos, por conta disso é recomendado que a atualização do esquema de vacinação seja realizada precocemente^3^ ^), (^ ^6^ ^), (^ ^7^ ^), (^ ^8^ ^), (^ ^9^ ^), (^ ^10^ ^), (^ ^11^. Apesar de a administração de algumas vacinas poder gerar um aumento transitório da carga viral, este evento não tem significância clínica e não pode ser um impeditivo para a vacinação^5^.

As PVHIV apresentam um risco aumentado de adquirir doenças imunopreveníveis e, uma vez que adquirem estas doenças, têm uma maior chance de evoluir para quadros mais graves. Por conta deste cenário, este público tem recomendação específica de vacinação^5^.

No Brasil, o Ministério da Saúde, através do Programa Nacional de Imunizações (PNI), oferece gratuitamente um calendário de vacinação especial, onde deve ser avaliado o estado imunológico do indivíduo e, caso não exista contraindicação, a recomendação é de que o esquema seja atualizado^1^ ^), (^ ^12^ ^), (^ ^13^.

Apesar de o Ministério da Saúde não realizar a avaliação rotineira da cobertura vacinal em indivíduos adultos, estima-se que a adesão ao calendário seja baixa neste público. Entre as PVHIV esta realidade não difere, e apesar da importância da vacinação nestes indivíduos, os estudos apontam baixas coberturas vacinais^6^ ^), (^ ^8^ ^), (^ ^10^ ^), (^ ^14^.

Um estudo desenvolvido nos EUA demonstrou que menos de 50% das PVHIV avaliadas estavam adequadamente vacinadas segundo as recomendações vigentes no país^8^. Em outro estudo desenvolvido na Bélgica, a cobertura da vacina hepatite B em PVHIV foi de 24,4% e contra o pneumococo foi de 72,6%^14^.

A decisão de vacinar ou não é influenciada por múltiplos fatores, que incluem pessoas, comportamentos e investimento; uma das formas de se motivar os indivíduos a se vacinarem é a recomendação feita pelo profissional de saúde^15^ ^), (^ ^16^.

O desconhecimento dos profissionais de saúde, em especial dos profissionais de enfermagem, em relação ao esquema de vacinação indicado para PVHIV e todos os fatores que envolvem a imunização deste público, provoca a falta ou insuficiência de recomendações a respeito da importância da vacinação. Além disso, colabora para a insegurança do indivíduo em se vacinar, sendo escassos os estudos que abordam esta temática^17^ ^), (^ ^18^.

Os profissionais de saúde, desde que munidos de informações adequadas e de qualidade em relação à imunização, podem fornecer orientações atualizadas e confiáveis, o que pode colaborar no aumento da confiança nas vacinas. Além disso, podem monitorar o comparecimento dos indivíduos à sala de vacinação e buscar aqueles em situação de atraso^10^ ^), (^ ^16^.

Para tanto, a educação permanente em saúde, por meio de ações educativas que acontecem no cotidiano do trabalho, se mostra uma importante estratégia para aumentar a capacidade resolutiva e a eficiência dos serviços de saúde com a possibilidade de causar mudanças no contexto social^19^ ^), (^ ^20^. Os profissionais de enfermagem precisam ser constantemente capacitados em relação aos conteúdos relacionados à imunização.

Diante do que foi exposto, este estudo objetivou analisar a efetividade de uma intervenção educativa no conhecimento de profissionais de enfermagem em relação à imunização de pessoas com o vírus da imunodeficiência humana.

## Método

### Delineamento do estudo

Trata-se de um estudo quase-experimental não randomizado, do tipo antes e depois, no qual foi realizada uma intervenção educativa online relacionada à imunização de PVHIV para profissionais de enfermagem. O texto deste manuscrito foi desenvolvido segundo as recomendações do SQUIRE 2.0.

### Local em que aconteceu a coleta de dados

O estudo foi realizado no município de Ribeirão Preto, São Paulo-SP, Brasil, a 312 km da capital, com uma população estimada de 711.825 habitantes. O município conta em sua rede pública com 50 unidades básicas de saúde (UBS), das quais 36 dispunham de sala de vacinas e cinco serviços especializados voltados para as pessoas que vivem com HIV/aids. Em relação aos serviços especializados, um deles tem sala de vacinas, três funcionam na mesma área física de UBS com salas de vacinas e um deles não tem sala de vacinas no mesmo espaço físico^21^.

### População e amostra

Foi encaminhado um *e-mail* aos supervisores das 36 unidades de saúde com sala de vacinas e dos cinco serviços especializados da rede pública do município informando o início da fase de intervenção, que se constituiu no curso de capacitação *online*. O *e-mail* solicitava aos supervisores que informassem o número de profissionais interessados em participar do estudo através da realização do curso e preenchimento de questionário aplicado antes e após o desenvolvimento deste curso.

Com base na informação do número de interessados, foram encaminhados às unidades os termos de consentimento livre e esclarecido (TCLE), os profissionais devolveram à pesquisadora o TCLE assinado e informando o *e-mail* para contato, para onde foram encaminhadas as orientações para matrícula e acesso ao curso de capacitação.

A amostragem deste estudo, portanto, foi intencional, constituída por profissionais de enfermagem (auxiliares/técnicos de enfermagem e enfermeiros) que aceitaram participar do estudo e que se enquadraram em um dos seguintes critérios de inclusão: atuar nas salas de vacinas do sistema público municipal de saúde, estar direta ou indiretamente envolvidos nas ações públicas municipais de imunização no momento do oferecimento do curso, ou atuar nos serviços especializados do Sistema Único de Saúde (SUS) do município do estudo onde as PVHIV realizam acompanhamento.

### Intervenção educativa

Foi desenvolvido um curso de capacitação *online*, por meio do Ambiente Virtual de Aprendizagem (AVA) Moodle, na modalidade curso de extensão, disponibilizado pela Universidade de São Paulo (USP), intitulado “Capacitação sobre imunização de pessoas que vivem com a infecção pelo vírus da imunodeficiência adquirida-HIV/aids”.

O curso, elaborado conforme as diretrizes do PNI^12^ e a literatura científica sobre o tema, era composto por quatro módulos. O programa educativo foi construído fundamentando-se nos princípios da andragogia, considerando as necessidades de aprendizagem relatadas por aqueles que seriam convidados a participar do curso com base na experiência da pesquisadora enquanto coordenadora do programa de imunização e enfermeira da vigilância epidemiológica e as dificuldades percebidas ao longo do tempo. Seguindo a proposta metodológica da andragogia, o processo de aprendizagem foi autodirigido, em clima informal e colaborativo entre educandos e educador^22^.

O curso foi oferecido de forma assíncrona entre agosto e novembro de 2021, teve duração total de três horas e ficou disponível para acesso conforme a disponibilidade do participante. O conteúdo foi dividido em quatro módulos: 1 - Importância da vacinação como prática de saúde pública/significado da promoção da saúde; 2 - Conceitos básicos em imunização; 3 - Vacinas atenuadas e inativadas-indicações para PVHIV; e 4 - Esquema vacinal para adultos infectados pelo HIV.

O curso foi composto por uma aula gravada, um fórum de discussão para dúvidas e material de apoio (referência bibliográfica e *slides* utilizados na aula) para facilitar a compreensão.

Conforme o cronograma proposto, a partir de dezembro/2021 não foi mais permitida a realização de matrículas no curso. Pelo fato de ser necessário que as aulas e os conteúdos sejam atualizados segundo as recomendações vigentes, o curso não foi mais disponibilizado.

### Instrumento utilizado para a coleta de informações

Os dados utilizados para avaliação do conhecimento foram coletados na própria plataforma Moodle onde o curso foi oferecido por meio de um questionário chamado de teste de conhecimento, elaborado com base nas diretrizes do PNI^12^ por profissional com ampla experiência na área de imunização. O instrumento de coleta de dados foi avaliado por seis especialistas com experiência profissional nas temáticas sobre imunização e HIV, sendo todos enfermeiros, mestres e doutores.

Os especialistas avaliaram o questionário quanto ao conteúdo, objetivo, linguagem verbal e relevância. O Índice de Validade de Conteúdo (IVC) foi calculado para cada item por meio de uma escala de cinco pontos, pelo conjunto de características da avaliação: conteúdo (9 itens; IVC=1,00), objetivo (5 itens; IVC=0,97), linguagem verbal (7 itens; IVC: 0,95), relevância (3 itens; IVC=1,00) e pelo instrumento total (24 itens; IVC=0,97). Todas as sugestões dos juízes foram acatadas.

O teste de conhecimento continha duas partes, uma com 10 questões referentes aos dados de identificação dos participantes e a outra com 35 questões referentes ao conhecimento técnico dos profissionais em relação à imunização das PVHIV. As questões que avaliavam o conhecimento técnico dos profissionais foram distribuídas em quatro categorias separadas conforme as temáticas abordadas nos módulos do curso de capacitação: I. Importância da vacinação como prática de saúde pública/significado da promoção da saúde (11 questões), II. Conceitos básicos em imunização (6 questões), III. Vacinas atenuadas e inativadas-indicações para PVHIV (8 questões), IV. Esquema vacinal para adultos infectados pelo HIV (10 questões). Foram utilizadas questões objetivas e para cada uma delas deveria ser escolhida uma das seguintes opções: “concordo”, “discordo” ou “não sei”, sendo apenas uma a resposta correta.

### Variáveis do estudo

As variáveis do estudo foram as relacionadas à identificação dos participantes - sexo, idade, local de trabalho, tempo de atuação e dados referentes à formação; e referentes ao conhecimento técnico em imunização de PVHIV abordadas em cada categoria de conhecimento avaliada.

### Coleta de dados

O curso de capacitação que compôs a intervenção educativa deste estudo foi realizado no período de agosto a novembro de 2021.

A coleta de dados ocorreu através do preenchimento do teste de conhecimento na própria plataforma onde o curso foi realizado, antes e após o desenvolvimento do curso de capacitação.

### Tratamento e análise dos dados

Após a finalização do curso de capacitação online, os dados foram extraídos da plataforma do curso em uma planilha do *Microsoft Office Excel* ^®^ e transportados para o programa IBM^®^ *Statistical Package for the Social Sciences* (SPSS^®^) *Statistics* versão 25, onde foi realizada a análise dos dados. Para o cálculo do “d” de Cohen foi utilizada a função cohen.d do pacote “effsize” do *software* estatístico R.

Os dados de identificação foram descritos por meio de distribuição de frequência (absoluta e relativa), mediana, média e desvio padrão. Para o teste de conhecimento, as respostas foram categorizadas em “correta” e “incorreta”, foi posteriormente realizada a contagem de quantas questões cada participante respondeu corretamente antes (variável Q1_acerto) e após a intervenção (variável Q2_acerto). Foi testada a normalidade das duas variáveis e apenas a variável Q1_acerto apresentou normalidade (teste Shapiro-Wilk p=0,194).

Para a avaliação de conhecimento dos participantes do curso, foram considerados os seguintes conceitos, determinados por intervalo de acertos de 20% - insuficiente (0┤20), regular (20┤40), bom (40┤60), ótimo (60┤80), excelente (80┤100), conforme proposto por outros autores^23^.

Os dados foram separados pelas categorias que compuseram o teste de conhecimento. Como não passaram pelo teste de normalidade, foram comparados pelo teste não paramétrico de Wilcoxon pareado para comparar a mediana de acertos antes e depois do curso de capacitação por questão, pelo número total de questões e por categoria do conhecimento.

Para as associações foram utilizados o coeficiente de correlação de Spearman (idade, tempo de atuação, tempo de formação), teste de Kruskal-Wallis de amostras independentes (unidade e distrito em que trabalha e maior formação) e teste U de Mann-Whitney de amostras independentes (função que exerce na Secretaria Municipal da Saúde/SMS e se recebeu treinamento em imunização), sendo os valores p<0,05 considerados com evidência estatística.

### Aspectos éticos

O projeto de pesquisa foi submetido à Comissão de Avaliação de Projetos de Pesquisa (CAPP) da SMS do município do estudo e posteriormente ao Comitê de Ética em Pesquisa (CEP) da Escola de Enfermagem de Ribeirão Preto (EERP) da USP, obtendo o parecer favorável (Parecer consubstanciado do CEP nº 4.782.341).

## Resultados

Após a divulgação do estudo nas unidades de saúde, 143 profissionais assinaram o TCLE e receberam orientações de matrícula no curso por *e-mail*. Dentre estes, 130 se matricularam na plataforma e 90 acessaram o conteúdo. Entretanto, 77 (100%) concluíram todas as etapas do curso, constituindo a amostra deste estudo.

A média de idade entre os participantes foi de 43,2 anos (min-max: 24-69; DP+/-8,2), 75 (97,4%) eram do sexo feminino e a faixa etária com maior frequência foi a de 40 a 49 anos (36; 46,7%) (Tabela 1).

**Tabela 1:** Distribuição dos participantes segundo sexo, faixa etária, formação e atuação profissional (n=77). Ribeirão Preto, SP, Brasil, 2021

Características	Participantes	
	f*	%
**Sexo**		
Masculino	2	2,6
Feminino	75	97,4
**Faixa etária (anos)**		
20 a 29	2	2,6
30 a 39	23	29,9
40 a 49	36	46,7
50 a 59	14	18,2
60 ou mais	2	2,6
**Unidade de saúde em que trabalha**		
Serviço especializado 1	5	6,5
Serviço especializado 2	6	7,8
Serviço especializado 3	2	2,6
Serviço especializado 4	1	1,3
Serviço especializado 5	3	3,9
UBS^†^/Unidade de Saúde da Família	45	58,4
Serviço de Urgência	6	7,8
Vigilância Epidemiológica	3	3,9
Outros	6	7,8
**Distrito em que trabalha**		
Leste	32	41,6
Central	15	19,5
Norte	10	13,0
Oeste	11	14,3
Sul	3	3,9
Sem distrito	6	7,8
**Tempo de atuação em sala de vacinas/serviço especializado (anos)**		
0 a 9	46	59,7
10 a 19	23	29,9
20 a 29	7	9,1
30 a 39	1	1,3
**Tempo de formação (anos)**		
0 a 9	8	10,4
10 a 19	37	48,0
20 a 29	22	28,6
30 a 39	10	13,0
**Função desenvolvida na SMS** ^‡^		
Auxiliar/Técnico de Enfermagem	42	54,5
Enfermeiro	34	44,2
Não é servidor da SMS^‡^	1	1,3
**Treinamento em imunização**		
Sim	52	67,5
Não	25	32,5

*f=Frequência; ^†^UBS=Unidade Básica de Saúde; ^‡^SMS=Secretaria Municipal de Saúde

Entre os participantes, 32 (41,6%) atuavam no distrito leste. Em relação ao local de trabalho, 17 (22,1%) trabalhavam nos serviços especializados que acompanham as PVHIV e 45 (58,4%) trabalhavam em UBS. No geral, 46 (59,7%) profissionais estavam vinculados em suas unidades há menos de 10 anos, com uma média de tempo no serviço de 7,9 anos (DP+/-7,7) (Tabela 1).

A média de tempo de formação foi de 18,4 anos (DP+/-7,4), com um mínimo de 2 e máximo de 35 anos de formação. Quase metade dos participantes (37; 48,0%) havia se formado entre 10 e 19 anos e 42 (54,5%) exerciam a função de auxiliar ou técnico de enfermagem na SMS. Entre os participantes, 52 (67,5%) haviam realizado algum treinamento em imunização anteriormente (Tabela 1).

Quanto ao conhecimento, a média de acertos passou de 22,6 (DP+/-4,3) no pré-teste para 26,6 (DP+/-4,3) após a realização do curso de capacitação e o desempenho melhorou com aumento na mediana de acertos de 23,0 para 27,0 (p<0,001).

No pré-teste, 38 participantes (49,4%) apresentaram um número de acertos inferior à mediana (23,0), sendo que no pós-teste 67 participantes (87,0%) atingiram um número de acertos igual ou superior a este índice.

O valor obtido do “d” de Cohen foi igual a - 0,94 (IC=[-1,277 ; -0.606]). Uma vez que o valor 0 não está incluído, há evidência, ao nível de confiança de 95%, de que há uma diferença significativa entre as médias, e conforme apresentado por Cohen^24^, o valor encontrado fornece evidência de que a magnitude do efeito dessa diferença é alta.

Na categoria 1- Importância da vacinação como prática de saúde pública/significado da promoção da saúde, cinco questões (45,4%) obtiveram um índice de acertos maior que 80% na fase pré-intervenção. Na fase pós-intervenção o conceito “excelente” foi atingido em oito questões (72,7%).

A média de acertos nesta categoria foi de 8,3 (DP+/-1,4) no pré-teste onde se observou aumento no pós-teste, com média de 9,2 acertos (DP+/-1,5) e a mediana de acertos passou de 8,0 para 9,0 (p<0,001).

O aumento no número de acertos ocorreu em oito questões (72,7%). Observou-se p<0,001 nas questões sobre quais profissionais podem indicar a vacinação para PVHIV (Q1) e sobre a necessidade de prescrição médica para administrar vacinas neste público (Q3). A questão sobre cobertura vacinal em adultos (Q6) teve p=0,007 e na sobre hesitação vacinal (Q8) observou-se p=0,002. Passaram do nível de conhecimento ótimo no pré-teste para o excelente no pós-teste as questões 1, 3 e 8; a questão 6 passou do nível regular para o bom.

As questões que apresentaram que é de conhecimento dos profissionais de saúde o fato do movimento antivacinista estar ganhando força no Brasil nos últimos anos, com aumento no número de acertos de 88,3% para 94,8% (Q7) e de 71,4% para 77,9% (Q10), apresentaram p=0,132 e p=0,225, respectivamente (Tabela 2).

**Tabela 2:** Comparação entre a porcentagem de acertos nas questões do teste de conhecimento na categoria 1 (Importância da vacinação como prática de saúde pública) antes e após a realização do curso de capacitação (n=11, 100%). Ribeirão Preto, SP, Brasil, 2021

Nº	Questões da categoria 1	Antes	Depois	p-valor*
		% de acertos	% de acertos	
Q1	O único profissional de saúde responsável por indicar vacinas às PVHIV^†^ é o infectologista. (D^‡^)	64,9	93,5	**<0,001**
Q2	As PVHIV^†^ têm recomendações de vacinação específicas pelo PNI^§^. (C^||^)	96,1	97,4	0,655
Q3	A equipe da Sala de Vacinas pode administrar nas PVHIV^†^ somente as vacinas que forem prescritas pelo médico infectologista que realiza o acompanhamento do paciente. (D^‡^)	66,2	94,8	**<0,001**
Q4	A busca ativa de indivíduos com esquema de vacinação em atraso pela equipe das Salas de Vacina e dos Serviços Especializados se constitui em uma importante ação para garantir a completude do esquema vacinal. (C^||^)	**100,0**	**96,1**	0,083
Q5	Algumas doenças já foram controladas, como o sarampo, assim, na avaliação de risco e benefício, não há a necessidade de administrar a vacina contra sarampo nas PVHIV^†^. (D^‡^)	85,7	89,6	0,366
Q6	O PNI^§^ disponibiliza vários imunizantes através do SUS^¶^ e o país mantém boas coberturas vacinais em indivíduos adultos. (D^‡^)	24,7	44,2	**0,007**
Q7	Muitos países enfrentam problemas com a recusa vacinal, um fenômeno complexo que envolve vários fatores, este fenômeno vem se estabelecendo no Brasil nos últimos anos. (C^||^)	88,3	94,8	0,132
Q8	O indivíduo que não concorda em completar o esquema vacinal, porém, aceita receber algumas vacinas pode ser considerado um hesitante quanto a vacinação. (C^||^)	76,6	93,5	**0,002**
Q9	À medida que aumenta o número de vacinas oferecidas e seu uso por meio de programas de vacinação, diminui a preocupação das pessoas com a segurança dos imunizantes e a desconfiança da necessidade de seu uso. (D^‡^)	**57,1**	**37,7**	**0,014**
Q10	Os movimentos antivacinais têm seu início no século XIX a partir da utilização da vacina contra a varíola, a primeira vacina desenvolvida, e vêm ganhando forças nos últimos anos por sua propagação nas mídias sociais. (C^||^)	71,4	77,9	0,225
Q11	A queda nas coberturas vacinais aumenta a incidência de doenças evitáveis, e, consequentemente, aumenta o número de mortes evitáveis, o que se constitui um risco para a saúde pública. (C^||^)	**100,0**	**98,7**	0,317

*O nível de significância é 0,05; ^†^PVHIV=Pessoas que vivem com o vírus da imunodeficiência humana; ^‡^D=Discordo; ^§^PNI=Programa Nacional de Imunizações; ^||^C=Concordo; ^¶^SUS=Sistema Único de Saúde

Sobre os conceitos básicos em imunização avaliados na categoria 2, duas questões (33,3%) obtiveram um índice de acertos maior que 80% na fase pré-intervenção. Na fase pós-intervenção, aumentou para três questões (50,0%) com o conceito “excelente”. A média de 3,6 acertos (DP+/-1,3) no pré-teste aumentou para 4,4 acertos (DP+/-1,3) no pós-teste e a mediana de acertos se manteve em 4,0 (p=<0,001).

Após a intervenção educativa, observou-se que quatro questões (66,7%) apresentaram um aumento no número de acertos. A questão que avaliava a composição das vacinas (Q13) teve p<0,001, passou do insuficiente no pré-teste para nível de acertos bom no pós-teste; e a questão que abordava a capacidade de resposta da vacina polissacarídica não conjugada (Q14) teve p<0,001 e passou de nível de conhecimento regular no pré-teste para excelente no pós-teste (Tabela 3).

**Tabela 3:** Comparação entre a porcentagem de acertos nas questões do teste de conhecimento na categoria 2 (Conceitos básicos em imunização) antes e após a realização do curso de capacitação (n=6, 100%). Ribeirão Preto, SP, Brasil, 2021

Nº	Questões da categoria 2	Antes	Depois	p-valor*
		% de acertos	% de acertos	
Q12	Vacinas combinadas são aquelas em que um produto imunologicamente menos potente é acrescentado a outro produto imunologicamente mais potente, conseguindo-se, dessa maneira, que o primeiro produto adquira características de potência imunológica que antes não possuía. (D^†^)	42,9	53,2	0,131
Q13	Vacina conjugada é aquela composta por dois ou mais antígenos de agentes infecciosos diferentes em uma única preparação. (D^†^)	18,2	44,2	**<0,001**
Q14	Nas vacinas inativadas polissacarídicas não conjugadas, a imunidade é de curta duração (três a cinco anos, em geral), pois a resposta imune não envolve a estimulação de linfócitos relacionados à imunidade celular. (C^‡^)	39,0	85,7	**<0,001**
Q15	Na triagem pré-vacinação devem ser investigadas possíveis contraindicações, por exemplo, o uso de antibióticos, que contraindica a maioria das vacinas. (D^†^)	89,6	94,8	0,248
Q16	Recomenda-se adiar a vacinação em caso de doença febril aguda moderada ou grave até a melhora do quadro. (C^‡^)	**98,7**	**97,4**	0,564
Q17	A ocorrência de eventos adversos deve ser notificada, qualquer evento adverso constitui contraindicação para doses futuras. (D^†^)	**74,0**	**68,8**	0,317

*O nível de significância é 0,05; ^†^D=Discordo; ^‡^C=Concordo

Para as questões que apresentaram a definição de vacinas combinadas (Q12) e situação de contraindicação para administração de vacinas (Q15), o aumento no número de acertos foi de 42,9% para 53,2% e 89,6% para 94,8%, com p=0,131 e p=0,248, respectivamente (Tabela 3).

Na categoria 3, avaliaram-se as vacinas atenuadas e inativadas-indicações para PVHIV. Identificou-se que quatro questões (50,0%) obtiveram um índice de acertos acima de 80% no pré-teste, e cinco (62,5%) no pós-teste.

No pré-teste, a média foi de 5,5 acertos (DP+/-1,3) nesta categoria e aumentou no pós-teste com média de 6,3 acertos (DP+/-1,4), a mediana passou de 6,0 para 7,0 (p<0,001).

O aumento no número de acertos ocorreu em cinco questões (62,5%). A questão sobre a composição da vacina contra a hepatite A (Q18) teve p=0,035, a sobre a administração de vacinas vivas atenuadas entre PVHIV (Q19) com p<0,001 e as questões sobre intervalo entre vacinas inativadas (Q21 e Q23) com p=0,050 e p=0,001, respectivamente (Tabela 4).

**Tabela 4:** Comparação entre a porcentagem de acertos nas questões do teste de conhecimento na categoria 3 (Vacinas atenuadas e inativadas - indicações para PVHIV*) antes e após a realização do curso de capacitação (n=8, 100%). Ribeirão Preto, SP, Brasil, 2021

Nº	Questões da categoria 3	Antes	Depois	p-valor^†^
		% de acertos	% de acertos	
Q18	A vacina contra hepatite A é composta por vírus inativados. (C^‡^)	85,7	94,8	**0,035**
Q19	As vacinas virais vivas atenuadas, preparadas com antígenos vivos, não podem ser administradas em PVHIV*. (D)	42,9	77,9	**<0,001**
Q20	As vacinas inativadas são produzidas a partir de microrganismos inteiros inativados ou partículas de microrganismos e não conseguem produzir doenças. (C^‡^)	**94,8**	**93,5**	0,317
Q21	Um usuário do serviço de saúde comparece para receber as vacinas Dupla Adulto e Tríplice Viral com encaminhamento do profissional do Serviço Especializado. A profissional da sala de vacinas verifica que o usuário recebeu uma dose de vacina meningocócica ACWY na rede privada há 10 dias e orienta o paciente a retornar em 20 dias, ao estar contraindicado fazer outras vacinas com intervalo inferior a 30 dias. (D^§^)	67,5	79,2	**0,050**
Q22	As vacinas inativadas geralmente não são contraindicadas para PVHIV*. (C^‡^)	**83,1**	**81,8**	0,819
Q23	É preciso respeitar 30 dias de intervalo entre as vacinas inativadas quando não aplicadas no mesmo dia. (D^§^)	61,0	84,4	**0,001**
Q24	As vacinas atenuadas somente podem ser administradas em PVHIV assintomáticas com a contagem de linfócitos T CD4>350 cel/mm^3^. (D^§^)	22,1	23,4	0,847
Q25	As vacinas contra COVID-19 que se comportam como vacinas inativadas podem ser administradas em PVHIV*. (C^‡^)	**96,1**	**94,8**	0,655

*PVHIV=Pessoas que vivem com o vírus da imunodeficiência humana; ^†^O nível de significância é 0,05; ^‡^C=Concordo; ^§^D=Discordo

A questão 18 se manteve no nível de conhecimento excelente, a questão 19 passou do nível bom no pré-teste para o ótimo no pós-teste, a questão 21 manteve o nível ótimo e a questão 23 passou do nível ótimo para o excelente (Tabela 4).

Destaca-se que a questão 24, que avaliou o conhecimento sobre administração de vacinas atenuadas em PVHIV assintomáticas com a contagem de linfócitos T (LT) CD4>350 cel/mm^3^ teve nível de conhecimento regular antes e após intervenção (Tabela 4).

Referente ao esquema vacinal para PVHIV adultas avaliado na categoria 4, das dez questões elaboradas, apenas uma (10,0%) obteve um índice de acertos superior a 80% no teste aplicado antes do curso de capacitação. No pós-teste foram três questões (30,0%) que obtiveram o conceito “excelente”.

A média de acertos no pré-teste foi de 5,2 acertos (DP+/-1,9) e 6,7 acertos (DP+/-1,6) no pós-teste, a mediana de acertos passou de 5,0 para 7,0 (p<0,001).

Observou-se aumento no número de acertos em nove questões (90,0%). Apenas a questão que abordava o esquema da vacina meningocócica C (Q31) apresentou o menor número de acertos tanto antes quanto após o curso de capacitação e, ainda, observou-se diminuição no número de acertos, com nível de conhecimento regular no pré-teste para insuficiente no pós-teste, com p=0,317 (Tabela 5).

**Tabela 5:** Comparação entre a porcentagem de acertos nas questões do teste de conhecimento na categoria 4 (Esquema vacinal para adultos infectados pelo vírus da imunodeficiência humana) antes e após a realização do curso de capacitação (n=10, 100%). Ribeirão Preto, SP, Brasil, 2021

Nº	Questões da categoria 4	Antes	Depois	p-valor*
		% de acertos	% de acertos	
Q26	A vacina contra Influenza é contraindicada para pessoas com linfócitos T CD4<200 células/mm^3^. (D^†^)	57,7	74,0	**0,009**
Q27	PVHIV^‡^ têm a recomendação de receber a vacina pneumocócica 23 com esquema de duas doses com intervalo de 5 anos entre elas. (C^§^)	72,7	93,5	**0,000**
Q28	É contraindicado administrar a vacina contra febre amarela em PVHIV^‡^. (D^†^)	54,5	81,8	**0,000**
Q29	A vacina pneumocócica 23 deve ser administrada nas PVHIV^‡^ a partir de 8 semanas da data de recebimento da vacina pneumocócica 13. (C^§^)	51,9	63,6	**0,050**
Q30	A vacina monovalente contra varicela está indicada para todas as PVHIV^‡^. (D^†^)	44,2	62,3	**0,011**
Q31	O esquema de vacinação com a vacina meningocócica C para PVHIV^‡^ é de duas doses com intervalo de 60 dias. (D^†^)	**23,4**	**18,2**	0,317
Q32	Após a conclusão do esquema de vacinação contra hepatite B em PVHIV^‡^ é recomendada a realização de sorologia para avaliar a soroconversão. Caso a sorologia tenha resultado negativo, recomenda-se repetir o esquema completo. (C^§^)	55,8	72,7	**0,024**
Q33	Vacinar os profissionais de saúde e os familiares das PVHIV^‡^ é uma forma de ampliar a proteção deste público. (C^§^)	93,5	100,0	**0,025**
Q34	O momento ideal para iniciar qualquer vacina para PVHIV^‡^ é 6 meses após o início da TARV^||^. (D^†^)	40,3	58,4	**0,013**
Q35	O esquema de vacinação contra HPV^¶^ em PVHIV^‡^ é de três doses (0, 2 e 6 meses) na faixa etária de 9 a 26 anos. (D^†^)	16,9	48,1	**0,000**

*O nível de significância é 0,05; ^†^D=Discordo; ^‡^PVHIV=Pessoas que vivem com o vírus da imunodeficiência humana; ^§^C=Concordo; ^||^TARV=Terapia Antirretroviral; ^¶^HPV=Papilomavírus Humano

Por outro lado, destaca-se que seis (60,0%) das questões desta categoria obtiveram nível de conhecimento bom no pré-teste, quatro delas (66,7%) atingiram o conceito excelente no pós-teste. Esta foi a categoria com maior aumento no nível de conhecimento entre os participantes depois da realização do curso de capacitação (Tabela 5).

A mediana de acertos apresentou melhora em todas as categorias avaliadas, todas com p<0,001, sendo maior nas categorias 1 (Importância da vacinação como prática de pública/significado da promoção da saúde), 3 (Vacinas atenuadas e inativadas - indicações para PVHIV) e 4 (Esquema vacinal para adultos infectados pelo HIV). Na categoria 2 (Conceitos básicos em imunização), a mediana de acertos permaneceu a mesma, porém, a média de acertos foi maior no pós-teste.

Na comparação com as características de trabalho e formação, o número maior de acertos apresentou associação com a unidade em que trabalha no pré-teste (p=0,038), com uma maior média de acertos entre os profissionais da Vigilância Epidemiológica (27,0) e os profissionais do Serviço Especializado 1 (26,0).

## Discussão

A intervenção educativa desenvolvida mostrou-se efetiva para melhorar o conhecimento dos profissionais de enfermagem acerca da imunização de PVHIV. Tal resultado é relevante e valida a utilização, pelos gestores, de intervenção semelhante como parte da educação continuada para aprimorar e desenvolver ampla base de conhecimento de profissionais de enfermagem sobre esta temática.

Na literatura, outro estudo também demonstrou que o curso de capacitação online breve e direcionado se mostrou efetivo para melhorar o conhecimento de conteúdos específicos relacionados à saúde e pode ser uma opção na prática clínica, colaborando na educação permanente^25^.

A educação permanente em saúde por meio de uma intervenção educativa direcionada aos profissionais de enfermagem mostrou-se uma estratégia eficaz para melhorar o conhecimento e a confiança destes profissionais em relação à imunização de PVHIV, o que pode impactar positivamente na cobertura vacinal deste público.

Cabe ressaltar que nenhum estudo na literatura foi encontrado sobre o contexto específico do conhecimento dos profissionais de saúde relacionado à imunização das PVHIV^17^. Um estudo realizado com enfermeiros no Catar identificou melhora no conhecimento sobre vacinação em enfermagem para a Atenção Primária à Saúde após um curso de capacitação, sendo encontradas lacunas importantes em diferentes aspectos^26^.

Os profissionais de enfermagem apresentavam um bom conhecimento na maioria dos aspectos avaliados, mesmo antes do desenvolvimento do curso de capacitação, porém identificaram-se várias lacunas, que puderam ser superadas após o desenvolvimento do curso com uma melhora na mediana dos acertos.

No teste de conhecimento, houve um aumento no número de acertos referente à questão que afirmava que somente o médico infectologista poderia indicar vacinas para PVHIV. Esta questão é importante, visto que qualquer profissional de saúde capacitado pode avaliar a situação vacinal e indicar vacinas, colaborando para o alcance das coberturas necessárias para o controle das doenças infecciosas, principalmente neste público com alto risco de complicações por estas doenças^12^ ^), (^ ^13^ ^), (^ ^14^ ^), (^ ^27^ ^), (^ ^28^ ^), (^ ^29^.

Houve aumento também no conhecimento sobre a necessidade de prescrição médica para vacinação das PVHIV. Segundo os protocolos, apenas as vacinas atenuadas necessitam de prescrição médica para este grupo, uma vez que estão contraindicadas na vigência de imunodepressão grave^12^ ^), (^ ^13^ ^), (^ ^27^ ^), (^ ^30^.

A maioria dos profissionais participantes deste estudo concordou que a hesitação vacinal vem se estabelecendo no Brasil nos últimos anos. Mais de 70%, tanto antes quanto após a realização do curso de capacitação, concordaram que o movimento antivacinista vem ganhando forças no país. No pré-teste, todos os participantes concordaram que a queda na cobertura vacinal é um risco para a saúde pública, uma vez que aumenta a ocorrência de doenças evitáveis e, consequentemente, o número de mortes.

A partir de 2016, as coberturas vacinais vêm caindo no Brasil para as crianças, e o país verifica um aumento na hesitação vacinal. Apesar de não realizar a avaliação da cobertura em indivíduos adultos, estima-se que este cenário também se reflita neste público. Em relação às PVHIV, não há uma avaliação efetiva da situação vacinal nos serviços onde realizam o seu acompanhamento^5^.

O crescimento do movimento antivacinista se deu principalmente após a campanha de vacinação contra a covid-19, com a disseminação de *fake news* e teorias conspiratórias, levando à desconfiança da população em relação aos imunizantes. Isto vem preocupando as autoridades sanitárias, pois a queda nas coberturas vacinais aumenta o risco de ocorrência e reintrodução de doenças controladas ou já erradicadas^10^ ^), (^ ^16^ ^), (^ ^31^ ^), (^ ^32^ ^), (^ ^33^ ^), (^ ^34^ ^), (^ ^35^.

Mais de 80% dos participantes deste estudo discordaram da afirmação de que não é necessário administrar vacinas para doenças já controladas, porém menos de 50% discordou de que a preocupação com a segurança das vacinas e a desconfiança em relação a elas diminuiu com o aumento no número de imunizantes oferecidos. Na realidade, à medida que aumenta o número de vacinas oferecidas e as doenças estão controladas, o receio em relação aos eventos adversos da vacina passa a ser maior do que o receio em relação à própria doença^34^ ^), (^ ^36^.

Menos da metade dos participantes deste estudo no pré-teste discordaram da afirmação de que o melhor momento para iniciar a vacinação em PVHIV é seis meses após o início da TARV; já no pós-teste, os que discordaram desta afirmação não chegaram a 60%, demonstrando a insegurança dos profissionais em vacinar este público.

Mesmo com a possibilidade de uma resposta vacinal reduzida na vigência de uma replicação viral não controlada ou com valores mais baixos de LT CD4, é recomendado que as vacinas sejam administradas conforme o calendário vigente. Devem, ainda, ser realizadas logo após o diagnóstico da infecção pelo HIV, assim que avaliadas as possíveis contraindicações para as vacinas atenuadas em função do status imunológico^2^ ^), (^ ^3^ ^), (^ ^12^ ^), (^ ^13^.

O Ministério da Saúde do Brasil não recomenda intervalos entre a coleta de exames e a administração de vacinas, apesar de alguns estudos recomendarem não medir a carga viral nas semanas seguintes à vacinação^12^ ^), (^ ^13^ ^), (^ ^37^.

Mais de 90% dos profissionais deste estudo concordaram que, tanto no pré-teste quanto no pós-teste, as vacinas inativadas não podem causar as doenças para as quais imunizam. Acima de 80% concordaram que estas vacinas geralmente não são contraindicadas para PVHIV. Apesar de as vacinas inativadas não serem contraindicadas para PVHIV, os profissionais podem ainda se sentir inseguros quanto à segurança destas vacinas neste público^12^ ^), (^ ^13^ ^), (^ ^28^ ^), (^ ^38^.

A quase totalidade dos participantes deste estudo concordou que as PVHIV têm recomendações específicas de vacinação, e que a complexidade do calendário para este público requer atualização, treinamento e capacitações constantes das equipes envolvidas.

Temas como a composição das vacinas geram dúvidas nos profissionais de saúde. Apesar de aumentar o número de acertos no pós-teste, a categoria 2 - Conceitos básicos em imunização continha a questão com um dos piores desempenhos no teste de conhecimento aplicado antes do curso de capacitação, a qual abordava a definição de vacina conjugada. Não compreender a composição das vacinas colabora para que os profissionais também não compreendam a recomendação em relação a cada esquema para diferentes imunobiológicos e a necessidade ou não de reforços.

Outra questão com baixo desempenho foi relacionada ao esquema da vacina meningocócica C para PVHIV, que no pós-teste ainda apresentou uma piora no número de acertos, demonstrando que mesmo depois do curso de capacitação os profissionais apresentavam dúvidas. Talvez durante o curso de capacitação, o esquema de vacinação não tenha sido apresentado claramente, assim como não estava claro no documento técnico disponibilizado pelo PNI naquele momento^12^. Este documento foi atualizado em 2023, a vacina meningocócica C foi substituída pela vacina meningocócica ACWY e o esquema de vacinação para este público está definido com duas doses com intervalo de oito semanas e reforços a cada cinco anos^13^.

Outro fator que dificulta a assimilação dos profissionais de saúde são esquemas diferentes para cada grupo ou faixa etária. Isso pode ser demonstrado também pelo desempenho dos profissionais no pré-teste em relação ao esquema da vacina contra o papilomavírus humano (HPV), a qual apresentou o pior resultado nesta fase do estudo.

A categoria do teste de conhecimento que abordava questões relativas à importância da vacinação na saúde pública foi a de melhor desempenho. Mostra-se, assim, que os profissionais das salas de vacinas e dos serviços que realizam o acompanhamento de PVHIV estão cientes do papel da imunização na prevenção de doenças e melhoria da qualidade de vida.

A categoria com pior desempenho foi a que abordava o esquema de vacinação para PVHIV, mostrando que mesmo após participarem do curso de capacitação ainda possuíam dúvidas a este respeito. Este fato reforça a importância da educação permanente em saúde para reduzir o déficit de conhecimento em relação aos calendários de vacinação para indivíduos adultos, principalmente pertencentes a grupos específicos da população. Uma maior familiarização com os esquemas de vacinação pela aplicação dos imunobiológicos no local onde os indivíduos realizam o seu acompanhamento pode contribuir para elevar os índices de vacinação^8^ ^), (^ ^9^ ^), (^ ^10^ ^), (^ ^16^ ^), (^ ^19^ ^), (^ ^28^ ^), (^ ^29^ ^), (^ ^31^ ^), (^ ^32^ ^), (^ ^33^ ^), (^ ^39^ ^), (^ ^40^ ^), (^ ^41^.

Apesar da queda nas coberturas vacinais sofrer interferência de múltiplos fatores, o conhecimento dos profissionais de saúde a respeito das vacinas indicadas para PVHIV, seus esquemas e suas contraindicações, podem impactar expressivamente neste indicador, ao auxiliar o indivíduo na tomada de decisão pela vacinação^34^ ^), (^ ^41^. Além disso, é uma temática pouco abordada na literatura, resultando em insegurança^17^.

Antes da realização do curso de capacitação, cerca de 22% dos profissionais discordaram de que as vacinas atenuadas somente poderiam ser aplicadas em PVHIV assintomáticas com o valor de LT CD4 acima de 350 células/mm^3^. O número de acertos nesta questão aumentou pouco no pós-teste, sendo esta a questão com o segundo pior desempenho nesta fase do estudo. Mesmo depois do desenvolvimento do curso, cerca de 20% dos profissionais acreditavam que as PVHIV não poderiam receber a vacina contra febre amarela.

Um estudo desenvolvido nos EUA demonstrou que a contagem de LT CD4 é um dos fatores associados à não adesão à vacinação das PVHIV^8^, apesar de ser conhecido que a administração de vacinas em PVHIV é uma estratégia segura, eficaz e importante neste grupo, especialmente as vacinas inativadas^28^. Conclui-se que não está claro para os profissionais de saúde qual é a condição que contraindica a imunização das PVHIV.

Em relação à vacina influenza inativada, recomendada para todas as PVHIV independentemente do status imunológico, especialmente indicadas para àquelas em imunodepressão, observou-se, no teste de conhecimento, um aumento no número de acertos referentes à indicação do imunizante às PVHIV, porém, no pós-teste, cerca de 25% ainda acreditavam que a vacina estaria contraindicada na vigência de imunodepressão grave.

Os profissionais de saúde precisam conhecer as verdadeiras contraindicações e as situações em que se recomenda o adiamento da vacinação para não colaborarem para a manutenção de baixas coberturas vacinais. Além disso, os médicos precisam estar atentos para prescrever as vacinas que somente podem ser administradas com a apresentação deste documento^8^ ^), (^ ^13^ ^), (^ ^27^.

Para garantir a proteção das PVHIV, principalmente entre aqueles com contraindicação temporária para receber alguma vacina, o PNI recomenda a administração de algumas vacinas para profissionais de saúde e para contatos domiciliares deste público^13^. Esta recomendação já era conhecida por 93% dos profissionais que participaram deste estudo no pré-teste. Após a participação no curso de capacitação, todos os profissionais concordaram que esta é uma estratégia importante.

As lacunas relacionadas à imunização devem ser combatidas desde a formação inicial do profissional de saúde, sendo necessário aumentar a carga horária relacionada à imunização, pois os indivíduos tendem a confiar na orientação de profissionais e das equipes de saúde^14^.

A avaliação da situação vacinal em todas as visitas do indivíduo no serviço de saúde, independentemente do atendimento médico, pode ser uma medida efetiva para melhorar a cobertura vacinal neste público, para tanto, estes profissionais precisam estar atualizados na temática e seguros quanto às suas orientações. Além disso, é necessário vigiar o comparecimento dos indivíduos no serviço de vacinação e realizar a busca daqueles que estão em atraso no esquema vacinal^8^ ^), (^ ^42^.

Os participantes deste estudo concordaram que a busca ativa é uma importante estratégia, mesmo antes da realização do curso de capacitação. Os motivos da não vacinação, que gera as baixas coberturas vacinais, precisam ser conhecidos tanto por gestores quanto por profissionais de saúde, para que medidas possam ser instituídas no enfrentamento deste problema crescente em todo o mundo^38^ ^), (^ ^39^.

Apesar da importância deste estudo para reforçar que os profissionais de saúde apresentam dúvidas em relação ao calendário de vacinação das PVHIV, o que pode impactar diretamente nas coberturas vacinais, é necessário apontar algumas limitações.

O oferecimento do curso de capacitação ocorreu em plena campanha nacional de vacinação contra a covid-19, momento de sobrecarga de trabalho para as equipes, o que impactou diretamente na adesão dos profissionais. Além disso, o curso apenas na modalidade online dificultou o acesso de profissionais de saúde não habilitados no uso desta tecnologia. Por outro lado, acredita-se que realizar o curso de forma assíncrona, na modalidade online, facilitou a participação de profissionais que não conseguiriam realiza-lo presencialmente naquele momento. Outro fator limitante é a seleção da amostra por conveniência, o que eventualmente pode ter gerado algum viés, uma vez que aqueles que aceitaram realizar o curso de capacitação poderiam se sentir mais confortáveis com a temática.

Apesar destas limitações, este estudo apresenta resultados robustos que reforçam a importância da educação permanente ao: garantir profissionais mais bem qualificados para avaliar a situação vacinal dos indivíduos, recomendar e administrar as vacinas indicadas nos protocolos do PNI, vigiar a completude do esquema vacinal e buscar pelos indivíduos em atraso no seu calendário de vacinação.

## Conclusão

Os dados deste estudo demonstraram que o conhecimento dos profissionais de enfermagem dos serviços onde as PVHIV realizam acompanhamento e das salas de vacinação pode ser insuficiente em vários aspectos, o que pode contribuir para a não indicação ou perda de oportunidades de vacinação.

O conhecimento dos profissionais de saúde, em especial dos profissionais de enfermagem, em relação às vacinas ofertadas, os esquemas de vacinação recomendados e as contra indicações na administração de imunobiológicos, é um dos fatores que pode influenciar diretamente a cobertura vacinal.

A atividade de educação permanente realizada através do oferecimento de um curso de capacitação online acerca da imunização de PVHIV se mostrou efetiva, visto que houve melhoria no conhecimento dos profissionais de enfermagem ao comparar os testes aplicados antes e após o desenvolvimento do curso. Além disso, o tamanho do efeito da intervenção foi considerado alto.

# Translation: Curso *online* sobre vacunación de personas con VIH/SIDA - efectividad sobre el conocimiento de los profesionales de enfermería*

**DOI:** 10.1590/1518-8345.7004.4277

**Authors:** Larissa Gerin, Elucir Gir, Lis Aparecida de Souza Neves, Luzia Márcia Romanholi Passos, Renato de Ávila Kfouri, Renata Karina Reis

**Affiliations:** 1 Secretaria Municipal da Saúde, Divisão de Vigilância Epidemiológica, Ribeirão Preto, SP, Brasil; 2 Universidade de São Paulo, Escola de Enfermagem de Ribeirão Preto, Centro Colaborador de la OPS/OMS para el Desarrollo de la Investigación en Enfermería, Ribeirão Preto, SP, Brasil; 3 Secretaria Municipal da Saúde, Departamento de Vigilância em Saúde, Ribeirão Preto, SP, Brasil; 4 Sociedade Brasileira de Imunizações, São Paulo, SP, Brasil

**Keywords:** Cobertura de Vacunación, Vacunación, VIH, Conocimiento, Enfermeras Practicantes, Educación en Salud

## Abstract

**Objetivo:**

analizar la efectividad de una intervención educativa sobre el conocimiento de los profesionales de enfermería con respecto a la inmunización de personas con el virus de la inmunodeficiencia humana.

**Método:**

estudio cuasiexperimental que evaluó los conocimientos de los profesionales mediante una evaluación de conocimiento aplicada antes y después del desarrollo de un curso de capacitación *online*. Los datos se analizaron mediante frecuencia, mediana, media, desviación estándar y pruebas de asociación.

**Resultados:**

la muestra estuvo compuesta por 77 profesionales de enfermería cuya edad promedio fue de 43,2 años (DE+/-8,2). Más de la mitad de los individuos trabajaba en unidades básicas de salud (58,4%), el 22,1% trabajaba en servicios especializados que realizan seguimiento clínico a personas portadoras del virus de la inmunodeficiencia humana, 42 (54,5%) trabajaban como auxiliar o técnico en enfermería. El desempeño de los profesionales mejoró después de la intervención, la mediana del número de respuestas correctas aumentó de 23,0 a 27,0 (p<0,001).

**Conclusión:**

dictar un curso de capacitación *online* sobre la inmunización de personas con el virus de la inmunodeficiencia humana, como actividad de educación continua, demostró ser eficaz para mejorar el conocimiento de los profesionales de enfermería sobre el tema.

## Destacados

**(1)** Los servicios no evalúan el estado de vacunación de las personas con VIH.

**(2)** El conocimiento de los profesionales de la salud puede influir en las tasas de vacunación.

**(3)** El conocimiento de los profesionales de la salud sobre inmunización puede no ser suficiente.

## Introducción

La vacunación es una importante estrategia de salud pública para prevenir enfermedades infecciosas en la población general. Con el paso de los años, además de controlar enfermedades como el sarampión y la polio, erradicó la viruela.

Las personas que viven con el virus de la inmunodeficiencia humana (PVVIH), que tras la llegada de la terapia antirretroviral (TARV) viven de manera similar a las personas no infectadas y, por lo tanto, están más expuestas, deben mantener actualizado su calendario de vacunación de acuerdo con las recomendaciones actuales para mejorar la calidad y la esperanza de vida^1^ ^), (^ ^2^ ^), (^ ^3^.

A pesar de la importancia que tiene para este público actualizar el calendario de vacunación, existe temor, tanto por parte de los profesionales de la salud como de esos individuos, respecto a la seguridad y eficacia de las vacunas administradas, principalmente por los riesgos de eventos adversos, según el grado de inmunosupresión. Además, las constantes actualizaciones del esquema y la inclusión de nuevos inmunizantes en el calendario de vacunación pueden generar dudas entre los profesionales de la salud, incluso en los profesionales de enfermería que trabajan en las salas de vacunación^4^ ^), (^ ^5^.

Postergar la administración de vacunas hasta que el sistema inmunológico de las PVVIH se reconstruya completamente puede aumentar el riesgo de estos individuos, por lo que se recomienda actualizar tempranamente el calendario de vacunación^3^ ^), (^ ^6^ ^), (^ ^7^ ^), (^ ^8^ ^), (^ ^9^ ^), (^ ^10^ ^), (^ ^11^. Si bien la administración de algunas vacunas puede generar un aumento transitorio de la carga viral, este evento no tiene significación clínica y no puede ser un impedimento para la vacunación^5^.

Las personas que viven con el VIH tienen un mayor riesgo de contraer enfermedades prevenibles mediante vacunas y, una vez que las adquieren, tienen mayores posibilidades de desarrollar cuadros más graves. Por ello, se recomienda específicamente la vacunación para ese público^5^.

En Brasil, el Ministerio de Salud, a través del Programa Nacional de Inmunizaciones (PNI), brinda un esquema de vacunación especial, gratuito, en el que hay que evaluar el estado inmunológico del individuo y, si no hay contraindicación, se recomienda actualizar el esquema^1^ ^), (^ ^12^ ^), (^ ^13^.

Si bien el Ministerio de Salud realiza evaluaciones de rutinaria de la cobertura de vacunación en adultos, se estima que el cumplimiento del esquema de esa población es bajo. Lo mismo ocurre con las PVVIH y, a pesar de la importancia que tiene la vacunación para esas personas, los estudios indican que tienen una baja cobertura de vacunación^6^ ^), (^ ^8^ ^), (^ ^10^ ^), (^ ^14^.

Un estudio realizado en EE.UU. demostró que menos del 50% de las PVVIH evaluadas estaban adecuadamente vacunadas según las recomendaciones vigentes en el país^8^. En otro estudio realizado en Bélgica, la cobertura de vacunación contra la hepatitis B en PVVIH fue del 24,4% y contra el neumococo del 72,6%^14^.

La decisión de vacunar o no se ve influenciada por múltiples factores, entre ellos las personas, los comportamientos y la inversión; una de las formas de motivar a las personas a vacunarse es la recomendación del profesional de la salud^15^ ^), (^ ^16^.

El desconocimiento de los profesionales de la salud, especialmente de enfermería, sobre el calendario de vacunación recomendado para las PVVIH y todos los factores que implican la inmunización de este público, llevan a la nula o escasa recomendación sobre la importancia de la vacunación, además contribuyen al miedo del individuo a vacunarse, y son pocos los estudios que abordan este tema^17^ ^), (^ ^18^.

Los profesionales de la salud, siempre que cuenten con información adecuada y de calidad sobre la inmunización, pueden brindar orientación actualizada y confiable, lo que puede ayudar a aumentar la confianza en las vacunas y, además, pueden monitorear la asistencia de las personas a la sala de vacunación y realizar una búsqueda activa de las que tienen el calendario desactualizado^10^ ^), (^ ^16^.

Para ello, la educación permanente en salud, a través de acciones educativas que se desarrollan diariamente en el trabajo, se presenta como una estrategia importante para aumentar la capacidad resolutiva y la eficiencia de los servicios de salud que puede provocar cambios en el contexto social^19^ ^), (^ ^20^. Los profesionales de enfermería deben estar constantemente capacitados sobre los contenidos relacionados con la inmunización.

Por lo tanto, este estudio tuvo como objetivo analizar la efectividad de una intervención educativa sobre el conocimiento de los profesionales de enfermería con respecto a la inmunización de personas con el virus de la inmunodeficiencia humana.

## Método

### Diseño del estudio

Se trata de un estudio no aleatorizado, cuasiexperimental, del tipo antes y después, en el que se realizó una intervención educativa *online* relacionada con la inmunización de PVVIH para profesionales de enfermería. El texto de este manuscrito fue desarrollado según las recomendaciones del SQUIRE 2.0.

### Lugar donde se realizó la recolección de datos

El estudio se realizó en el municipio de Ribeirão Preto, São Paulo-SP, Brasil, a 312 km de la capital, con una población estimada de 711.825 habitantes. El municipio cuenta en su red pública con 50 unidades básicas de salud (UBS), de las cuales 36 contaban con salas de vacunación, y cinco servicios especializados dirigidos a personas que viven con VIH/SIDA. En cuanto a los servicios especializados, uno de ellos cuenta con sala de vacunación, tres funcionan en la misma área física de la UBS con salas de vacunación y uno de ellos no cuenta con sala de vacunación en el mismo espacio físico^21^.

### Población y muestra

Se envió un correo electrónico a los supervisores de las 36 unidades de salud con salas de vacunación y de los 5 servicios especializados de la red pública del municipio informándoles sobre el inicio de la fase de intervención, que consistía en el curso de capacitación *online*. El correo electrónico les solicitaba a los supervisores que informaran la cantidad de profesionales interesados en participar en el estudio mediante la realización del curso y la cumplimentación de un cuestionario aplicado antes y después del desarrollo del este curso.

Luego de obtener el número de interesados, se enviaron los términos de consentimiento libre e informado (TCLI) a las unidades, los profesionales le devolvieron el TCLI firmado a la investigadora y proporcionaron el correo electrónico de contacto, al que se enviaron las indicaciones para la inscripción y el acceso al curso de capacitación.

La muestra del presente estudio fue intencional y estuvo conformada por profesionales de enfermería (auxiliares/técnicos en enfermería y enfermeros) que aceptaron participar en el estudio y que cumplieron uno de los siguientes criterios de inclusión: trabajar en las salas de vacunación del sistema público municipal de salud, estar directa o indirectamente involucrado en acciones públicas municipales de inmunización al momento del dictado del curso o trabajar en servicios especializados del Sistema Único de Salud (SUS) en el municipio del estudio donde se realiza el seguimiento de las personas que viven con VIH.

### Intervención educativa

Fue desarrollado un curso de capacitación *online*, por medio del Entorno Virtual de Aprendizaje (EVA) *Moodle*, en la modalidad curso de extensión, dictado por la Universidad de São Paulo (USP), titulado “*Capacitação sobre imunização de pessoas que vivem com a infecção pelo vírus da imunodeficiência adquirida - HIV/aids*” (Capacitación en inmunización de personas que viven con infección por el virus de inmunodeficiencia adquirida- VIH/SIDA).

El curso constaba de cuatro módulos y fue elaborado según las directrices del PNI^12^ y la literatura científica sobre el tema. El programa educativo se elaboró en base a los principios de la andragogía, considerando las necesidades de aprendizaje informadas por quienes serían invitados a participar del curso a partir de la experiencia de la investigadora como coordinadora del programa de inmunizaciones y enfermera de vigilancia epidemiológica y las dificultades percibidas a lo largo del tiempo. Siguiendo la propuesta metodológica de la andragogía, el proceso de aprendizaje fue autodirigido, en un ambiente informal y colaborativo entre los estudiantes y el educador^22^.

El curso se dictó de forma asíncrona entre agosto y noviembre de 2021, tuvo una duración total de tres horas y estuvo disponible para que los participantes accedieran en función de su disponibilidad. El contenido se dividió en cuatro módulos: 1 - Importancia de la vacunación como práctica de salud pública/significado de la promoción de la salud, 2 - Conceptos básicos sobre inmunización, 3 - Vacunas atenuadas e inactivadas - indicaciones para PVVIH y 4 - Calendario de vacunación para adultos infectados por VIH.

El curso constó de una clase grabada, un foro de discusión de dudas y material de apoyo (referencias bibliográficas y diapositivas utilizadas en la clase) para facilitar la comprensión.

De acuerdo con el cronograma propuesto, a partir de diciembre de 2021 ya no se permitía la inscripción al curso. Debido a que era necesario actualizar las clases y los contenidos en función de las recomendaciones vigentes, el curso ya no está disponible.

### Instrumento utilizado para la recolección de información

Los datos utilizados para evaluar los conocimientos fueron recolectados en la plataforma Moodle, donde se dictó el curso a través de un cuestionario denominado prueba de conocimiento, elaborada según las directrices del PNI^12^ por un profesional con amplia experiencia en el área de inmunización. El instrumento de recolección de datos fue evaluado por seis expertos con experiencia profesional en los temas de inmunización y VIH, todos eran enfermeros, másteres y doctores.

Los expertos evaluaron el contenido, el objetivo, el lenguaje verbal y la relevancia del cuestionario. Para cada ítem se calculó el Índice de Validez de Contenido (IVC) mediante una escala de cinco puntos, a partir del conjunto de características de evaluación: contenido (9 ítems; IVC=1,00), objetivo (5 ítems; IVC=0,97), lenguaje verbal (7 ítems; IVC: 0,95), relevancia (3 ítems; IVC=1,00) y del instrumento total (24 ítems; IVC=0,97). Todas las sugerencias de los jueces fueron aceptadas.

La prueba de conocimiento costaba de dos partes, una con 10 preguntas referidas a los datos de identificación de los participantes y otra con 35 preguntas referidas al conocimiento técnico de los profesionales sobre la inmunización de las PVVIH. Las preguntas que evaluaban el conocimiento técnico de los profesionales estaban distribuidas en cuatro categorías separadas según los temas tratados en los módulos del curso de capacitación: I. Importancia de la vacunación como práctica de salud pública/significado de la promoción de la salud (11 preguntas), II. Conceptos básicos sobre inmunización (6 preguntas), III. Vacunas atenuadas e inactivadas: indicaciones para personas que viven con el VIH (8 preguntas), IV. Calendario de vacunación para adultos infectados por el VIH (10 preguntas). Se utilizaron preguntas objetivas y para cada una de ellas debían elegir una de las siguientes opciones: “de acuerdo”, “en desacuerdo” o “no sé”, solo había una respuesta correcta.

### Variables del estudio

Las variables de estudio fueron las relacionadas con la identificación de los participantes: género, edad, lugar de trabajo, tiempo de experiencia y datos relativos a la formación; y los relacionados con el conocimiento técnico sobre inmunización de las personas que viven con el VIH abordado en cada categoría de conocimiento evaluada.

### Recolección de dados

El curso de capacitación que conformó la intervención educativa de este estudio se realizó de agosto a noviembre de 2021.

La recolección de datos se produjo mediante la realización de la prueba de conocimiento en la plataforma donde se realizó el curso, antes y después del desarrollo del curso de capacitación.

### Procesamiento y análisis de datos

Después de completar el curso de capacitación *online*, los datos se extrajeron de la plataforma del curso en una hoja de cálculo de *Microsoft Office Excel* ^®^ y se exportaron al programa estadístico IBM^®^ *Statistical Package for the Social Sciences* (SPSS^®^) versión 25, donde se llevó a cabo el análisis de los datos. Para calcular la “d” de Cohen se utilizó la función cohen.d del paquete “effsize” del *software* estadístico R.

Los datos de identificación se describieron mediante distribución de frecuencia (absoluta y relativa), mediana, media y desviación estándar. Para la prueba de conocimiento, las respuestas se categorizaron en “correctas” e “incorrectas”, luego de lo cual se contabilizó el número de preguntas que cada participante respondió correctamente antes (variable P1_correcta) y después de la intervención (variable P2_correcta). Se probó la normalidad de las dos variables y solo la variable P1_correcta mostró normalidad (prueba de Shapiro-Wilk p=0,194).

Para evaluar el conocimiento de los participantes del curso, se consideraron los siguientes conceptos, determinados por un intervalo de respuestas correctas del 20%: insuficiente (0┤20), regular (20┤40), bueno (40┤60), muy bueno (60┤80), excelente (80┤100), según lo propuesto por otros autores^23^.

Los datos fueron separados por las categorías que conformaron la prueba de conocimiento. Como no superaron la prueba de normalidad, la comparación se realizó mediante la prueba no paramétrica de Wilcoxon pareada para comparar la mediana de las respuestas correctas antes y después del curso de capacitación por pregunta, por número total de preguntas y por categoría de conocimiento.

Para las asociaciones se utilizó el coeficiente de correlación de Spearman (edad, tiempo de experiencia, tiempo de egresado), prueba de Kruskal-Wallis para muestras independientes (unidad y distrito en el que trabaja y mayor nivel de formación) y prueba U de Mann-Whitney para muestras independientes (función que desempeña en la Secretaría Municipal de Salud/SMS y si recibió capacitación sobre inmunización), los valores de p<0,05 se consideraron evidencia estadística.

### Aspectos éticos

El proyecto de investigación fue presentado al Comité de Evaluación de Proyectos de Investigación (CAPP) de la SMS del municipio del estudio y posteriormente al Comité de Ética en Investigación (CEP) de la Escuela de Enfermería de Ribeirão Preto (EERP) de la USP, y obtuvo dictamen favorable (Dictamen consustanciado del CEP n.º 4.782.341).

## Resultados

Luego de la difusión del estudio en las unidades de salud, 143 profesionales firmaron el consentimiento libre e informado y recibieron instrucciones para la inscripción al curso por correo electrónico. De ellos, 130 se registraron en la plataforma y 90 accedieron al contenido. Sin embargo, fueron 77 (100%) los que completaron todas las etapas del curso y constituyeron la muestra del presente estudio.

La edad promedio de los participantes era de 43,2 años (mín-máx: 24-69; DE+/-8,2), 75 (97,4%) eran del sexo femenino y la franja etaria más frecuente fue la de 40 a 49 años (36; 46,7%) (Tabla 1).

**Tabla 1:** Distribución de los participantes según sexo, franja etaria, tiempo de egresado y actividad profesional (n=77). Ribeirão Preto, SP, Brasil, 2021

Características	Participantes	
	f*	%
**Sexo**		
Masculino	2	2,6
Femenino	75	97,4
**Franja etaria (años)**		
20 a 29	2	2,6
30 a 39	23	29,9
40 a 49	36	46,7
50 a 59	14	18,2
60 o más	2	2,6
**Unidad de salud en la que trabaja**		
Servicio especializado 1	5	6,5
Servicio especializado 2	6	7,8
Servicio especializado 3	2	2,6
Servicio especializado 4	1	1,3
Servicio especializado 5	3	3,9
UBS^†^/Unidad de Salud de la Familia	45	58,4
Servicio de Emergencia	6	7,8
Vigilancia Epidemiológica	3	3,9
Otras	6	7,8
**Distrito en el que trabaja**		
Este	32	41,6
Centro	15	19,5
Norte	10	13,0
Oeste	11	14,3
Sur	3	3,9
Sin distrito	6	7,8
**Tiempo de trabajo en la sala de vacunación/Servicio especializado (a**ñ**os)**		
0 a 9	46	59,7
10 a 19	23	29,9
20 a 29	7	9,1
30 a 39	1	1,3
**Tempo de egresado (a**ñ**os)**		
0 a 9	8	10,4
10 a 19	37	48,0
20 a 29	22	28,6
30 a 39	10	13,0
Función que realiza en la SMS^‡^		
Auxiliar/Técnico en enfermería	42	54,5
Enfermero	34	44,2
No trabaja en la SMS^‡^	1	1,3
**Capacitación sobre inmunización**		
Sí	52	67,5
No	25	32,5

*f=Frecuencia; ^†^UBS=Unidad Básica de Salud; ^‡^SMS=Secretaría Municipal de Salud

Del total de participantes, 32 (41,6%) trabajaban en el distrito este. En cuanto al lugar de trabajo, 17 (22,1%) trabajaban en los servicios especializados que realizan seguimiento a las personas que viven con el VIH y 45 (58,4%) trabajaban en la UBS. En total, 46 (59,7%) profesionales llevaban menos de 10 años trabajando en sus unidades, con una antigüedad media de 7,9 años (DE+/-7,7) (Tabla 1).

El tiempo medio de egresados era de 18,4 años (DE+/-7,4), con un mínimo de 2 y un máximo de 35 años de egresados. Casi la mitad de los participantes (37; 48,0%) habían egresado hace entre 10 y 19 años y 42 (54,5%) trabajaban como auxiliares o técnicos en enfermería en la SMS. Los participantes que habían completado previamente alguna formación en vacunación fueron 52 (67,5%) (Tabla 1).

En cuanto a los conocimientos, el promedio de respuestas correctas pasó de 22,6 (DE+/-4,3) en la prueba inicial a 26,6 (DE+/-4,3) al finalizar el curso de capacitación y el rendimiento mejoró con un aumento en la mediana del número de respuestas correctas de 23,0 a 27,0 (p<0,001).

En la prueba inicial, 38 participantes (49,4%) obtuvieron un número de respuestas correctas inferior a la mediana (23,0), y en la prueba posterior, 67 participantes (87,0%) lograron un número de respuestas correctas igual o superior a ese índice.

El valor obtenido para la “d” de Cohen fue igual a -0,94 (IC=[-1,277; -0,606]). Al no incluirse el valor 0, existe evidencia, según el nivel de confianza del 95%, de que existe una diferencia significativa entre las medias, y tal como lo presenta Cohen^24^, el valor obtenido proporciona evidencia de que la magnitud del efecto de esta diferencia es alta.

En la categoría 1 - Importancia de la vacunación como práctica de salud pública/significado de la promoción de la salud, cinco preguntas (45,4%) se obtuvo una tasa de respuestas correctas superior al 80% en la fase preintervención. En la fase postintervención, ocho preguntas (72,7%) alcanzaron un nivel “excelente”.

El promedio de respuestas correctas en esta categoría fue de 8,3 (DE+/-1,4) en la prueba inicial, y se observó un aumento en la prueba posterior con un promedio de 9,2 respuestas correctas (DE+/-1,5) y la mediana de respuestas correctas pasó de 8,0 a 9,0 (p<0,001).

El aumento en el número de respuestas correctas se produjo en ocho preguntas (72,7%). Se observó p<0,001 en las preguntas sobre qué profesionales pueden recomendarles la vacunación a las personas que viven con el VIH (P1) y sobre la necesidad de prescripción médica para administrarle vacunas a esta población (P3). La pregunta sobre cobertura de vacunación en adultos (P6) tuvo p=0,007 y la pregunta sobre reticencia a vacunarse (P8) tuvo p=0,002. Las preguntas 1, 3 y 8 pasaron de un nivel de conocimiento muy bueno en la prueba inicial a excelente en la prueba posterior; la pregunta 6 pasó de nivel regular a bueno.

Las preguntas que mencionaban que los profesionales de la salud saben que el movimiento antivacunas viene ganando fuerza en Brasil en los últimos años, tuvo un aumento en el número de respuestas correctas del 88,3% al 94,8% (P7) y del 71,4% al 77,9% (P10), y presentaron p=0,132 y p=0,225, respectivamente (Tabla 2).

**Tabla 2:** Comparación entre el porcentaje de respuestas correctas en las preguntas de la prueba de conocimiento de la categoría 1 (Importancia de la vacunación como práctica de salud pública) antes y después de finalizar el curso de capacitación (n=11, 100%). Ribeirão Preto, SP, Brasil, 2021

Nº	Preguntas de la categoría 1	Antes	Después	p-valor*
		% de resp. correctas	% de resp. correctas	
P1	El único profesional de la salud responsable de recomendar vacunas a las PVVIH^†^ es el especialista en enfermedades infecciosas. (D^‡^)	64,9	93,5	**<0,001**
P2	Hay recomendaciones de vacunación específicas en el PNI^§^ para las PVVIH^†^. (A^||^)	96,1	97,4	0,655
P3	El equipo de la Sala de Vacunación solo puede administrarles a las PVVIH^†^ las vacunas prescritas por el médico infectólogo que realiza el seguimiento del paciente. (D^‡^)	66,2	94,8	**<0,001**
P4	La búsqueda activa de personas con calendario de vacunación desactualizado por parte del equipo de Salas de Vacunación y Servicios Especializados constituye una acción importante para garantizar que se complete el calendario de vacunación. (A^||^)	**100,0**	**96,1**	0,083
P5	Algunas enfermedades ya han sido controladas, como el sarampión, por ende, al evaluar riesgo y beneficio, no es necesario administrar la vacuna contra el sarampión a las PVVIH^†^. (D^‡^)	85,7	89,6	0,366
P6	El PNI^§^ pone a disposición varias vacunas a través del SUS^¶^ y el país cuenta con una buena cobertura de vacunación en adultos. (D^‡^)	24,7	44,2	**0,007**
P7	Muchos países enfrentan problemas por la reticencia a las vacunas, un fenómeno complejo que involucra varios factores, este fenómeno se ha establecido en Brasil en los últimos años. (A^||^)	88,3	94,8	0,132
P8	Una persona que no acepta completar el esquema de vacunación, pero acepta recibir algunas vacunas, puede ser considerada reacia a vacunarse. (A^||^)	76,6	93,5	**0,002**
P9	A medida que aumenta el número de vacunas disponibles y la aplicación a través de programas de vacunación, disminuyen las preocupaciones de las personas sobre la seguridad de las vacunas y la desconfianza sobre la necesidad de las mismas. (D^‡^)	**57,1**	**37,7**	**0,014**
P10	Los movimientos antivacunas comenzaron en el siglo XIX con el uso de la vacuna contra la viruela, la primera vacuna desarrollada, y han ido ganando fuerza en los últimos años debido a su difusión en las redes sociales. (A^||^)	71,4	77,9	0,225
P11	La reducción de la cobertura de vacunación aumenta la incidencia de enfermedades prevenibles y, por ende, aumenta el número de muertes evitables, lo que constituye un riesgo para la salud pública. (A^||^)	**100,0**	**98,7**	0,317

*El nivel de significancia es del 0,05; ^†^PVVIH=Personas que viven con el virus de la inmunodeficiencia humana; ^‡^D=En desacuerdo; ^§^PNI=Programa Nacional de Inmunizaciones; ^||^A=De acuerdo; ^¶^SUS=Sistema Único de Salud

Con respecto a los conceptos básicos sobre inmunización evaluados en la categoría 2, dos preguntas (33,3%) obtuvieron una tasa de respuestas correctas superior al 80% en la fase preintervención. En la fase postintervención aumentó a tres preguntas (50,0%) con un nivel “excelente”. El promedio de 3,6 respuestas correctas (DE+/-1,3) en la prueba inicial pasó a 4,4 respuestas correctas (DE+/-1,3) en la prueba posterior y la mediana del número de respuestas correctas se mantuvo en 4,0 (p=<0,001).

Después de la intervención educativa, se observó que cuatro preguntas (66,7%) mostraron un aumento en el número de respuestas correctas. La pregunta que evaluó la composición de las vacunas (P13) tuvo p<0,001, y pasó de insuficiente en la prueba inicial al nivel de respuestas correctas bueno en la prueba posterior; y la pregunta que abordaba la capacidad de respuesta de la vacuna de polisacáridos no conjugados (P14) tuvo p<0,001 y pasó de un nivel de conocimiento regular en la prueba inicial a excelente en la prueba posterior (Tabla 3).

**Tabla 3:** Comparación entre el porcentaje de respuestas correctas en las preguntas de la prueba de conocimientos de la categoría 2 (Conceptos básicos sobre inmunización) antes y después de finalizar el curso de capacitación (n=6, 100%). Ribeirão Preto, SP, Brasil, 2021

Nº	Preguntas de la categoría 2	Antes	Después	p-valor*
		% de resp. correctas	% de resp. correctas	
P12	Las vacunas combinadas son aquellas en las que un producto con menor potencia inmunológica se añade a otro producto con mayor potencia inmunológica para lograr que el primer producto adquiera características de potencia inmunológica que antes no poseía. (D^†^)	42,9	53,2	0,131
P13	Una vacuna conjugada es aquella compuesta por dos o más antígenos de diferentes agentes infecciosos en una única preparación. (D^†^)	18,2	44,2	**<0,001**
P14	En las vacunas inactivadas de polisacáridos no conjugados, la inmunidad es de corta duración (de tres a cinco años, en general), ya que la respuesta inmune no implica la estimulación de los linfocitos relacionados con la inmunidad celular. (A^‡^)	39,0	85,7	**<0,001**
P15	En el triaje previo a la vacunación hay que investigar posibles contraindicaciones, por ejemplo, el uso de antibióticos, contraindicados para la aplicación de la mayoría de las vacunas. (D^†^)	89,6	94,8	0,248
P16	Se recomienda posponer la vacunación en casos de fiebre aguda moderada o grave hasta que mejore la condición. (A^‡^)	**98,7**	**97,4**	0,564
P17	Hay que informar la aparición de eventos adversos, cualquier evento adverso constituye una contraindicación para futuras dosis. (D^†^)	**74,0**	**68,8**	0,317

*El nivel de significancia es del 0,05; ^†^D=En desacuerdo; ^‡^A=De acuerdo

Para las preguntas que presentaban la definición de vacunas combinadas (P12) y la situación en la que se contraindica la administración de vacunas (P15), el número de respuestas correctas aumentó del 42,9% al 53,2% y del 89,6% al 94,8%, con p=0,131 y p=0,248, respectivamente (Tabla 3).

En la categoría 3 se evaluaron vacunas atenuadas e inactivadas - indicaciones para PVVIH. Se identificó que cuatro preguntas (50,0%) tuvieron una tasa de respuestas correctas superior al 80% en la prueba inicial y cinco (62,5%) en la prueba posterior.

En la prueba inicial el promedio fue de 5,5 respuestas correctas (DE+/-1,3) en esta categoría y aumentó en la prueba posterior con un promedio de 6,3 respuestas correctas (DE+/-1,4), la mediana aumentó de 6,0 a 7,0 (p<0,001).

Se produjo un aumento del número de respuestas correctas en cinco preguntas (62,5%). La pregunta sobre la composición de la vacuna contra la hepatitis A (P18) tuvo p=0,035, la pregunta sobre la administración de vacunas vivas atenuadas a las PVVIH (P19) tuvo p<0,001 y las preguntas sobre el intervalo entre vacunas inactivadas (P21 y P23) tuvo p=0,050 y p=0,001, respectivamente (Tabla 4).

**Tabla 4:** Comparación entre el porcentaje de respuestas correctas en las preguntas de la prueba de conocimiento de la categoría 3 (Vacunas atenuadas e inactivadas - indicaciones para PVVIH*) antes y después de completar el curso de capacitación (n=8, 100%). Ribeirão Preto, SP, Brasil, 2021

Nº	Preguntas de la categoría 3	Antes	Después	p-valor^†^
		% de resp. correctas	% de resp. correctas	
P18	La vacuna contra la hepatitis A contiene virus inactivados. (A^‡^)	85,7	94,8	**0,035**
P19	No se les pueden administrar vacunas de virus vivos atenuados preparadas con antígenos vivos a las PVVIH*. (D)	42,9	77,9	**<0,001**
P20	Las vacunas inactivadas se producen a partir de microorganismos inactivados completos o partículas de microorganismos y no son capaces de producir enfermedades. (A^‡^)	**94,8**	**93,5**	0,317
P21	Un usuario del servicio de salud derivado por profesional del servicio especializado se presenta para recibir la vacuna Doble Adulto y Triple Viral. El profesional de la sala de vacunación verifica que el usuario recibió una dosis de la vacuna meningocócica ACWY en la red privada hace 10 días y le aconseja al paciente regresar en 20 días, ya que está contraindicado recibir otras vacunas con un intervalo menor a 30 días. (D^§^)	67,5	79,2	**0,050**
P22	Las vacunas inactivadas generalmente no están contraindicadas para PVVIH*. (A^‡^)	**83,1**	**81,8**	0,819
P23	Es necesario respetar un intervalo de 30 días entre vacunas inactivadas cuando no se administren el mismo día. (D^§^)	61,0	84,4	**0,001**
P24	Las vacunas atenuadas sólo se pueden administrar a PVVIH* asintomáticas y con recuento de linfocitos T CD4>350 células/mm^3^. (D^§^)	22,1	23,4	0,847
P25	Se pueden administrar vacunas contra el COVID-19 que se comportan como vacunas inactivadas a PVVIH^*^. (A^‡^)	**96,1**	**94,8**	0,655

*PVVIH=Personas que viven con el virus de la inmunodeficiencia humana; ^†^El nivel de significancia es del 0,05; ^‡^A=De acuerdo; ^§^D=En desacuerdo

La pregunta 18 se mantuvo en un nivel de conocimiento excelente, la pregunta 19 pasó de un nivel bueno en la prueba inicial a un nivel muy bueno en la prueba posterior, la pregunta 21 mantuvo un nivel muy bueno y la pregunta 23 pasó de un nivel muy bueno a uno excelente (Tabla 4).

Cabe destacar que la pregunta 24, que evaluó el conocimiento sobre la administración de vacunas atenuadas a PVVIH asintomáticas con recuento de linfocitos T (LT) CD4>350 células/mm^3^, obtuvo un nivel de conocimiento regular antes y después de la intervención (Tabla 4).

En lo que respecta al calendario de vacunación de las PVVIH evaluadas en la categoría 4, de las diez preguntas elaboradas, sólo una (10,0%) obtuvo una tasa de respuestas correctas superior al 80% en la prueba aplicada antes del curso de capacitación, en la prueba posterior fueron tres las preguntas (30,0%) que obtuvieron un nivel “excelente”.

El promedio de respuestas correctas en la prueba previa fue de 5,2 (DE+/-1,9) y en la prueba posterior fue de 6,7 (DE+/-1,6), la mediana de respuestas correctas pasó de 5,0 a 7,0 (p<0,001).

Se observó que hubo un aumento en el número de respuestas correctas en nueve preguntas (90,0%). Sólo la pregunta que abordaba el calendario de vacunación contra el meningococo C (P31) presentó el menor número de respuestas correctas tanto antes como después del curso de capacitación y, además, se observó que registró una disminución en el número de respuestas correctas, el nivel de conocimiento en la prueba inicial fue regular y en la prueba posterior fue insuficiente, con p=0,317 (Tabla 5).

**Tabla 5:** Comparación entre el porcentaje de respuestas correctas en las preguntas de la prueba de conocimientos de la categoría 4 (Calendario de vacunación para adultos infectados por el VIH) antes y después de finalizar el curso de capacitación (n=10, 100%). Ribeirão Preto, SP, Brasil, 2021

Nº	Preguntas de la categoría 4	Antes	Después	p-valor*
		% de resp. correctas	% de resp. correctas	
P26	La vacuna contra la influenza está contraindicada para personas con linfocitos T CD4<200 células/mm^3^. (D^†^)	57,7	74,0	**0,009**
P27	Se recomienda que las PVVIH^‡^ reciban la vacuna neumocócica 23 con un esquema de dos dosis con un intervalo de 5 años entre ambas. (A^§^)	72,7	93,5	**0,000**
P28	Está contraindicado administrar la vacuna contra la fiebre amarilla a PVVIH^‡^. (D^†^)	54,5	81,8	**0,000**
P29	La vacuna neumocócica 23 debe ser administrada a las PVVIH^‡^ luego de 8 semanas a partir de la fecha de aplicación de la vacuna neumocócica 13. (A^§^)	51,9	63,6	**0,050**
P30	La vacuna monovalente contra la varicela está indicada para todas las PVVIH^‡^. (D^†^)	44,2	62,3	**0,011**
P31	El calendario de vacunación de la vacuna meningocócica C para PVVIH^‡^ es de dos dosis con 60 días de diferencia. (D^†^)	**23,4**	**18,2**	0,317
P32	Después de completar el esquema de vacunación contra la hepatitis B en las PVVIH^‡^, se recomienda realizar la serología para evaluar la seroconversión. Si los resultados de la serología son negativos, se recomienda repetir el esquema completo. (A^§^)	55,8	72,7	**0,024**
P33	Vacunar a profesionales de la salud y familiares de las PVVIH^‡^ es una forma de aumentar la protección de esta población. (A^§^)	93,5	100,0	**0,025**
P34	El momento ideal para comenzar a aplicar cualquier vacuna a las PVVIH^‡^ es 6 meses después de iniciar la TARV^||^. (D^†^)	40,3	58,4	**0,013**
P35	El esquema de vacunación contra el VPH^¶^ para las PVVIH^‡^ es de tres dosis (0, 2 y 6 meses) en la franja etaria de 9 a 26 años. (D^†^)	16,9	48,1	**0,000**

*El nivel de significancia es del 0,05; ^†^D=En desacuerdo; ^‡^PVVIH=Personas que viven con el virus de la inmunodeficiencia humana; ^§^A=De acuerdo; ^||^TARV=Terapia Antirretroviral; ^¶^VPH=Virus del Papiloma Humano

Por otro lado, cabe destacar que seis (60,0%) de las preguntas de esta categoría lograron un nivel de conocimiento bueno en la prueba inicial, cuatro de ellas (66,7%) alcanzaron un nivel excelente en la prueba posterior. Esta fue la categoría en la que los participantes registraron un mayor incremento en el nivel de conocimiento después de finalizar el curso de capacitación (Tabla 5).

La mediana del número de respuestas correctas mejoró en todas las categorías evaluadas, todas con p<0,001, y fue mayor en las categorías 1 (Importancia de la vacunación como práctica pública/significado de la promoción de la salud), 3 (Vacunas atenuadas e inactivadas - indicaciones para PVVIH) y 4 (Calendario de vacunación para adultos infectados por el VIH). En la categoría 2 (Conceptos básicos sobre inmunización), la mediana de respuestas correctas se mantuvo igual, pero la media de respuestas correctas fue mayor en la prueba posterior.

Al comparar las características laborales y de formación, el mayor número de respuestas correctas en la prueba inicial (p=0,038) se asoció con la unidad en la que trabajan, los profesionales de Vigilancia Epidemiológica (27,0) y los Profesionales de servicios especializados 1 (26,0) obtuvieron un mayor promedio de respuestas correctas.

## Discusión

La intervención educativa desarrollada resultó eficaz para mejorar el conocimiento de los profesionales de enfermería sobre la inmunización de las PVVIH. Este resultado es relevante y valida el uso, por parte de los gestores, de una intervención similar como parte de la educación continua para mejorar y desarrollar una amplia base de conocimientos de los profesionales de enfermería sobre este tema.

En la literatura, otro estudio también demostró que el curso de capacitación *online*, breve y específico, resultó eficaz para mejorar el conocimiento de contenidos específicos relacionados con la salud y puede ser una opción en la práctica clínica y contribuir a la educación continua^25^.

La educación continua en salud a través de una intervención educativa dirigida a profesionales de enfermería demostró ser una estrategia efectiva para mejorar el conocimiento y la confianza de estos profesionales sobre la inmunización de las PVVIH, lo que puede tener un impacto positivo en la cobertura de vacunación de esa población.

Cabe señalar que no se encontró ningún estudio en la literatura sobre el contexto específico del conocimiento de los profesionales de la salud relacionado con la inmunización de las PVVIH^17^. Un estudio realizado con enfermeros en Qatar identificó una mejora en el conocimiento sobre vacunación en enfermería para la Atención Primaria de Salud después de que realizaron un curso de capacitación y detectó lagunas importantes en diferentes aspectos^26^.

Los profesionales de enfermería tenían un buen nivel de conocimiento en la mayoría de los aspectos evaluados, incluso antes de realizar el curso de capacitación, pero se identificaron varias lagunas, que pudieron ser superadas después del desarrollo del curso con una mejora en la mediana del número de respuestas correctas.

En la prueba de conocimiento, hubo un aumento en el número de respuestas correctas a la pregunta que afirmaba que sólo un médico especialista en enfermedades infecciosas podría recomendar vacunas para las PVVIH. Este tema es importante, ya que cualquier profesional de la salud capacitado puede evaluar el estado de vacunación y recomendar vacunas, lo que contribuye a lograr la cobertura necesaria para controlar las enfermedades infecciosas, especialmente en esta población que tiene alto riesgo de complicaciones por esas enfermedades^12^ ^), (^ ^13^ ^), (^ ^14^ ^), (^ ^27^ ^), (^ ^28^ ^), (^ ^29^.

También aumentó el conocimiento sobre la necesidad de prescripción médica para la administración de vacunas a las PVVIH. Según los protocolos, para este grupo sólo las vacunas atenuadas requieren prescripción médica, ya que están contraindicadas en presencia de inmunosupresión grave^12^ ^), (^ ^13^ ^), (^ ^27^ ^), (^ ^30^.

La mayoría de los profesionales que participó en este estudio coincidió en que la reticencia a las vacunas se ha establecido en Brasil en los últimos años. Más del 70%, tanto antes como después de finalizar el curso de capacitación, coincidió en que el movimiento antivacunas ha ido ganando fuerza en el país. En la prueba inicial, todos los participantes coincidieron en que la disminución en la cobertura de vacunación es un riesgo para la salud pública ya que aumenta la aparición de enfermedades prevenibles y, por ende, el número de muertes.

Desde 2016, la cobertura de vacunación infantil en Brasil ha ido disminuyendo y el país ha experimentado un aumento de la reticencia a las vacunas. A pesar de que no se hizo una evaluación de la cobertura en individuos adultos, se estima que este fenómeno también se da en esa población. En lo que respecta a las PVVIH, no hay una evaluación efectiva del estado de vacunación en los servicios donde se les realiza el seguimiento^5^.

Con el crecimiento del movimiento antivacunas, especialmente después de la campaña de vacunación contra el COVID-19, debido a la difusión de noticias falsas y teorías conspirativas, la desconfianza de la población en los agentes inmunizantes preocupa a las autoridades sanitarias, ya que la disminución de la cobertura de vacunación aumenta el riesgo de aparición y reintroducción de enfermedades controladas o ya erradicadas^10^ ^), (^ ^16^ ^), (^ ^31^ ^), (^ ^32^ ^), (^ ^33^ ^), (^ ^34^ ^), (^ ^35^.

Más del 80% de los participantes en este estudio estuvo en desacuerdo con la afirmación sobre que no es necesario administrar vacunas para enfermedades que ya están controladas, pero menos del 50% estuvo en desacuerdo con que la preocupación por la seguridad de las vacunas y la desconfianza en ellas ha disminuido con el aumento del número de vacunas disponibles. En realidad, a medida que aumenta el número de vacunas disponibles y las enfermedades están controladas, el miedo a los eventos adversos de la vacuna se vuelve mayor que el miedo a la enfermedad en sí^34^ ^), (^ ^36^.

Menos de la mitad de los participantes en este estudio en la prueba inicial estuvo en desacuerdo con la afirmación sobre que el mejor momento para iniciar la vacunación de las PVVIH es seis meses después de iniciar la TARV, y en la prueba posterior los que estuvieron en desacuerdo con esta afirmación no llegaron al 60%, lo que demuestra la inseguridad que tienen los profesionales a la hora de vacunar a este público.

Incluso si es posible que la respuesta a la vacuna sea menor en presencia de replicación viral no controlada o con valores de LT CD4 más bajos, se recomienda que las vacunas se administren según el calendario vigente, poco después del diagnóstico de infección por VIH, una vez evaluadas las posibles contraindicaciones de las vacunas atenuadas en función del estado inmunológico^2^ ^), (^ ^3^ ^), (^ ^12^ ^), (^ ^13^.

El Ministerio de Salud de Brasil no recomienda intervalos entre la realización de pruebas y la administración de vacunas, aunque algunos estudios recomiendan no medir la carga viral en las semanas posteriores a la vacunación^12^ ^), (^ ^13^ ^), (^ ^37^.

Más del 90% de los profesionales de este estudio coincidieron, tanto en la prueba inicial como en la prueba posterior, en que las vacunas inactivadas no son capaces de provocar las enfermedades contra las cuales fueron formulas. Más del 80% estuvo de acuerdo en que estas vacunas generalmente no están contraindicadas para las PVVIH. Aunque las vacunas inactivadas no están contraindicadas para las PVVIH, los profesionales aún pueden sentirse inseguros sobre la seguridad de esas vacunas para dicha población^12^ ^), (^ ^13^ ^), (^ ^28^ ^), (^ ^38^.

Casi todos los participantes de este estudio coincidieron en que las PVVIH tienen recomendaciones específicas de vacunación, y que la complejidad del calendario para este público requiere actualización, entrenamiento y capacitación constante de los equipos en cuestión.

Temas como la composición de las vacunas les generan dudas a los profesionales de la salud. A pesar de que aumentó el número de respuestas correctas en la prueba posterior, la categoría 2 - Conceptos básicos sobre inmunización tenía la pregunta con uno de los peores resultados en la prueba de conocimiento aplicada antes del curso de capacitación, que abordó la definición de vacuna conjugada. El hecho de no entender la composición de las vacunas hace que los profesionales tampoco entiendan la recomendación con respecto a cada esquema de distintos inmunobiológicos y si es necesario o no aplicar refuerzos.

Otra pregunta con bajo desempeño fue la relacionado con el calendario de vacunación contra el meningococo C para PVVIH, que, en la prueba posterior, incluso, obtuvo un menor número de respuestas correctas, eso demuestra que, aun después de haber realizado el curso de capacitación, los profesionales tenían dudas. Quizás durante el curso de capacitación no se presentó claramente el calendario de vacunación y no estaba claro en el documento técnico que emitió el PNI en ese momento^12^. El documento fue actualizado en 2023, la vacuna meningocócica C fue reemplazada por la vacuna meningocócica ACWY y el calendario de vacunación para dicha población está definido con dos dosis con un intervalo de 8 semanas y refuerzos cada 5 años^13^.

Otro factor que dificulta la asimilación de los profesionales de la salud es que haya diferentes esquemas para cada grupo o franja etaria, lo que también se puede demostrar con el desempeño de los profesionales en la prueba inicial sobre el calendario de vacunación contra el virus del papiloma humano (VPH), que presentó el peor resultado en esa fase del estudio.

La categoría de la prueba de conocimiento que abordó cuestiones relacionadas con la importancia de la vacunación en la salud pública fue la que obtuvo el mejor desempeño, ello destaca que los profesionales de las salas de vacunación y de los servicios que llevan a cabo el seguimiento de las PVVIH son conscientes del papel de la inmunización para la prevención de enfermedades y para mejorar la calidad de vida.

La categoría con peor desempeño fue la que abordó el calendario de vacunación para PVVIH, lo que revela que aun después de participar en el curso de capacitación seguían teniendo dudas al respecto. Este hecho refuerza la importancia de la educación permanente en salud para reducir el déficit de conocimientos sobre los calendarios de vacunación de personas adultas, fundamentalmente de los que pertenecen a grupos poblacionales específicos. Un mayor conocimiento sobre los esquemas de vacunación con la aplicación de inmunobiológicos en el lugar donde se lleva a cabo el seguimiento de los pacientes puede contribuir al aumento de las tasas de vacunación^8^ ^), (^ ^9^ ^), (^ ^10^ ^), (^ ^16^ ^), (^ ^19^ ^), (^ ^28^ ^), (^ ^29^ ^), (^ ^31^ ^), (^ ^32^ ^), (^ ^33^ ^), (^ ^39^ ^), (^ ^40^ ^), (^ ^41^.

Si bien la disminución de la cobertura de vacunación está influenciada por múltiples factores, el conocimiento de los profesionales de la salud sobre las vacunas recomendadas para las PVVIH, los calendarios y las contraindicaciones, puede tener un impacto significativo en ese indicador, ya que ayuda al individuo a tomar la decisión de vacunarse^34^ ^), (^ ^41^. Además, es un tema poco discutido en la literatura, lo que genera inseguridad^17^.

Antes de realizar la capacitación, alrededor del 22% de los profesionales estaba en desacuerdo con que las vacunas atenuadas sólo se podían aplicar a las PVVIH asintomáticas y con un valor de LT CD4 superior a 350 células/mm^3^. El número de respuestas correctas a esta pregunta aumentó ligeramente en la prueba posterior, y es la segunda pregunta con peor desempeño en esa etapa del estudio. Incluso después del curso, alrededor del 20% de los profesionales creía que las PVVIH no podían recibir la vacuna contra la fiebre amarilla.

Un estudio realizado en EE.UU. demostró que el recuento de LT CD4 es uno de los factores asociados a la no adherencia a la vacunación de las PVVIH^8^, a pesar de que se sabe que la administración de vacunas a las PVVIH es una estrategia segura, eficaz e importante para ese grupo, especialmente las vacunas inactivadas^28^. Se concluye que no queda claro para los profesionales de la salud cuál es la condición que contraindica la inmunización de las PVVIH.

En lo que respecta a la vacuna inactivada contra la influenza, recomendada para todas las PVVIH, independientemente del estado inmunológico, e indicada especialmente para aquellas con inmunosupresión, en la prueba de conocimiento hubo un aumento en el número de respuestas correctas sobre la indicación del inmunizante para las PVVIH, sin embargo, después de la prueba, alrededor del 25% todavía creía que la vacuna estaba contraindicada en caso de inmunosupresión grave.

Los profesionales de la salud deben conocer las verdaderas contraindicaciones y situaciones en las que se recomienda posponer la vacunación para que no contribuyan a mantener las bajas coberturas de vacunación. Además, los médicos deben tener cuidado al prescribir vacunas que sólo pueden administrarse con la presentación de este documento^8^ ^), (^ ^13^ ^), (^ ^27^.

Para garantizar la protección de las personas que viven con el VIH, especialmente de aquellas con contraindicaciones temporales para recibir una vacuna, el PNI recomienda la administración de algunas vacunas a los profesionales de la salud y a quienes conviven con las PVVIH^13^. El 93% de los profesionales que participaron en este estudio en la prueba inicial ya conocía esa recomendación. Luego de participar en el curso de capacitación, todos los profesionales coincidieron en que se trata de una estrategia importante.

Las brechas relacionadas con la inmunización deben ser abordadas desde la formación inicial de los profesionales de la salud y es necesario aumentar la carga horaria relacionada con la inmunización, ya que los individuos tienden a confiar en la orientación de los profesionales y del equipo de salud^14^.

Evaluar el estado de vacunación en todas las visitas que realiza el individuo al servicio de salud, independientemente de la atención médica brindada, puede ser una medida eficaz para mejorar la cobertura de vacunación de esa población, por lo tanto, los profesionales deben estar actualizados en el tema y estar seguros sobre las orientaciones que les van a dar a los pacientes. Además, es necesario monitorear la asistencia de las personas al servicio de vacunación y realizar una búsqueda activa de los que tienen desactualizado el calendario de vacunación^8^ ^), (^ ^42^.

Los participantes de este estudio coincidieron en que la búsqueda activa es una estrategia importante, incluso antes de realizar el curso de capacitación. Tanto los directivos como los profesionales de la salud deben conocer las razones por la cuales las personas no se vacunan, que llevan a una baja cobertura de vacunación, para poder implementar medidas para combatir este creciente problema en todo el mundo^38^ ^), (^ ^39^.

Si bien este estudio es importante para destacar que los profesionales de la salud tienen dudas sobre el calendario de vacunación de las PVVIH, lo que puede tener un impacto directo en la cobertura de vacunación, es necesario señalar algunas limitaciones.

La capacitación fue impartida durante la campaña nacional de vacunación contra el COVID-19, momento en el que los equipos estaban sobrecargados de trabajo, lo que tuvo un impacto directo en la adherencia de los profesionales. Además, el hecho de que el curso se haya desarrollado solamente de forma *online* dificultó el acceso a los profesionales de la salud que no estaban calificados en el uso de esa tecnología. Por otro lado, se cree que realizar el curso de forma asincrónica *online* facilitó la participación de los profesionales que no podían realizar el curso en ese momento de forma presencial. Otro factor limitante es la selección de la muestra por conveniencia, lo que puede haber generado algún sesgo, ya que quienes aceptaron realizar el curso de capacitación podrían haberse sentido más cómodos con el tema.

A pesar de las limitaciones, este estudio presenta resultados sólidos que refuerzan la importancia de la educación continua que garantiza profesionales mejor calificados para evaluar el estado de vacunación de las personas, recomendar y administrar las vacunas indicadas en los protocolos del PNI, monitorear que el calendario de vacunación esté completo y realizar una búsqueda activa de las personas que tienen el calendario de vacunación desactualizado.

## Conclusión

Los datos de este estudio demostraron que el conocimiento de los profesionales de enfermería en los servicios en los que se realiza el seguimiento de las PVVIH y en las salas de vacunación puede no ser suficiente en varios aspectos, lo que puede contribuir a que no indiquen las vacunas o pierdan la oportunidad de vacunar a esas personas.

El conocimiento de los profesionales de la salud, especialmente de enfermería, sobre las vacunas disponibles, los calendarios de vacunación recomendados y las contraindicaciones en la administración de inmunobiológicos, es uno de los factores que puede influir directamente en la cobertura de vacunación.

La actividad de educación continua realizada a través de un curso de capacitación *online* sobre la inmunización de PVVIH resultó efectiva, dado que mejoró el conocimiento de los profesionales de enfermería, como se puede ver al comparar las pruebas aplicadas antes y después del dictado del curso, además, el tamaño del efecto de la intervención se consideró alto.
